# Multimodal integration of carotid plaque ultrasound radiomics and dynamic systemic immune-inflammation trajectories for subacute risk stratification after stroke thrombectomy: development and internal validation of an exploratory prognostic model

**DOI:** 10.3389/fneur.2026.1896641

**Published:** 2026-10-27

**Authors:** Haijuan Liu, Zhuangzhuang Lu, Li Xie, Fuzhen Miao, Jianhua Li, Huaibin Chen

**Affiliations:** 1 Department of Ultrasound Medicine, Liaocheng People’s Hospital, Liaocheng, Shandong, China; 2 Department of Emergency Neurosurgical Intensive Care Unit, Qilu Hospital of Shandong University, Jinan, Shandong, China; 3 Department of Ultrasound Medicine, Liaocheng No.3 People’s Hospital, Liaocheng, Shandong, China; 4 Department of Neurosurgery, Liaocheng People’s Hospital, Liaocheng, Shandong, China

**Keywords:** acute ischemic stroke, endovascular thrombectomy, machine learning, prognosis, radiomics, systemic immune-inflammation index

## Abstract

**Background:**

We hypothesized that integrating macroscopic carotid plaque architecture, quantified via ultrasound radiomics, with downstream micro-cellular temporal dynamics, defined by longitudinal immune trajectories, would yield a highly accurate prognostic framework for 90-day functional outcomes in patients undergoing endovascular thrombectomy (EVT).

**Methods:**

This single-center, retrospective cohort study consecutively included 316 AIS-LVO patients who underwent successful EVT (modified Thrombolysis in Cerebral Infarction score ≥2b) within 24 h of symptom onset. Patients were partitioned into a discovery (*N* = 221) and an internal validation cohort (*N* = 95) using stratified random sampling. To capture temporal inflammatory heterogeneity, the Systemic Immune-Inflammation Index (SII) was measured at days 0, 1, 3, and 7, and clustered using Latent Class Mixed Models (LCMM). Concurrently, high-dimensional radiomic features strictly adhering to the Image Biomarker Standardisation Initiative (IBSI) guidelines were extracted from baseline two-dimensional carotid ultrasound images. A radiomics score (Rad-score) was constructed utilizing Least Absolute Shrinkage and Selection Operator (LASSO) regression. Four progressive predictive models were developed, culminating in an Extreme Gradient Boosting (XGBoost) multimodal architecture. The primary endpoint was an unfavorable 90-day functional outcome (modified Rankin Scale score 3 to 6).

**Results:**

An unfavorable outcome occurred in 166 of 316 patients (52.5%). The LCMM objectively identified three longitudinal SII phenotypes. The “persistent high-inflammation” trajectory (Class 3, 17.0% of patients) was independently associated with an increased risk of an unfavorable outcome compared to the “rapid resolution” trajectory (Class 1) [adjusted odds ratio (aOR), 4.85; 95% confidence interval [CI], 2.20 to 10.85; *p* < 0.001]. The ultrasound Rad-score, comprising 9 core textural features, also demonstrated robust independent predictive value (aOR, 1.52; 95% CI, 1.21 to 1.92 per 0.1-point increase; *p* < 0.001). In the strictly isolated internal validation cohort, the full multimodal XGBoost model achieved an area under the receiver operating characteristic curve (AUC) of 0.92 (95% CI, 0.86 to 0.97), with an accuracy of 86.3%, sensitivity of 88.0%, and specificity of 84.4%. This performance was statistically superior to the standard clinical baseline model (AUC, 0.74; 95% CI, 0.63 to 0.84; *p* < 0.001 via DeLong test). Furthermore, the multimodal paradigm demonstrated clinical reclassification utility, yielding a continuous net reclassification improvement of 0.985 (95% CI, 0.684 to 1.286) and an integrated discrimination improvement of 0.194 (95% CI, 0.128 to 0.260).

**Conclusion:**

An exploratory model integrating baseline carotid plaque ultrasound radiomics with Day-0-to-Day-7 systemic immune-inflammation trajectories was associated with 90-day functional outcome after EVT and, on internal validation, discriminated outcomes more strongly than a conventional clinical model. Because the immune-trajectory component is only complete at Day 7, the model is intended for subacute post-EVT risk stratification rather than acute treatment selection, and requires external, prospective validation before any clinical application.

## 1 Introduction

Acute ischemic stroke (AIS) secondary to large vessel occlusion (LVO) accounts for approximately 38% of all ischemic stroke presentations and remains a predominant cause of global neurological morbidity and mortality (1, 2). Endovascular thrombectomy (EVT) has fundamentally transformed the management of AIS-LVO. According to comprehensive meta-analyses of pivotal clinical trials, EVT achieves successful macroscopic angiographic reperfusion [defined as a modified Thrombolysis in Cerebral Infarction (mTICI) score ≥ 2b] in 71 to 88% of patients (3). Despite this high technical success rate, nearly 40 to 50% of successfully recanalized patients fail to achieve functional independence (modified Rankin Scale [mRS] score 0–2) at 90 days (4). This clinical discrepancy, termed “futile recanalization,” represents a major therapeutic bottleneck, necessitating the development of precise, individualized prognostic stratification tools.

The pathophysiology driving futile recanalization is critically mediated by ischemia–reperfusion injury and the subsequent systemic thrombo-inflammatory cascade (5). Mechanical reperfusion triggers endothelial dysfunction, blood–brain barrier breakdown, and an infiltration of peripheral innate immune cells, culminating in secondary microvascular “no-reflow” phenomena (6, 7). The Systemic Immune-Inflammation Index (SII), a composite biomarker integrating peripheral platelet, neutrophil, and lymphocyte counts, accurately quantifies the systemic balance between pro-inflammatory/pro-thrombotic activation and acute stroke-induced immune suppression (8). Retrospective multicenter analyses demonstrate that a singular, elevated baseline SII (e.g., > 843 × 10^9/L) independently predicts 90-day poor functional outcomes with an odds ratio (OR) of 2.83 (9). However, the post-stroke systemic immune response is characterized by severe temporal heterogeneity. Conventional static, single-timepoint baseline assessments fail to capture the dynamic magnitude and duration of the inflammatory storm (10). Utilizing latent class mixed models (LCMM) to map longitudinal SII trajectories over the acute post-operative phase provides a superior, data-driven phenotypic quantification of immune responses (11).

Crucially, the downstream thrombo-inflammatory microenvironment is inherently linked to the upstream embolic source. Carotid atherosclerosis is the primary etiology for anterior circulation AIS-LVO (12). The structural vulnerability of the upstream carotid plaque—specifically the presence of a lipid-rich necrotic core and intraplaque hemorrhage—directly dictates the histological composition, rigidity, and inflammatory potential of the dislodged embolus (13). High-throughput radiomics enables the extraction of sub-visual, quantitative textural features (e.g., Gray Level Co-occurrence Matrix and Run Length Matrix) from standard two-dimensional B-mode carotid ultrasound images. Recent investigations indicate that ultrasound radiomic signatures can identify high-risk vulnerable plaques with an area under the receiver operating characteristic curve (AUC) of 0.82, significantly outperforming traditional morphological assessments (14). Nevertheless, the synergistic prognostic efficacy of integrating upstream macroscopic radiomic architectures with downstream systemic longitudinal immune trajectories remains uninvestigated (15).

To address this critical methodological and clinical gap, we propose a novel multimodal prognostic framework. We hypothesized that a machine-learning architecture integrating carotid plaque ultrasound radiomics with LCMM-derived longitudinal SII trajectories would yield a model with greater discrimination than conventional clinical metrics. Using a retrospective cohort of 316 AIS-LVO patients undergoing EVT, this study aimed to develop and internally validate an exploratory Extreme Gradient Boosting (XGBoost) model for subacute (Day-7) risk stratification of 90-day functional outcome, and to characterise the association between upstream plaque architecture and downstream systemic immune trajectories, rather than to provide an acute, bedside decision-support tool.

## 2 Methods

### 2.1 Study design, ethical approval, and participant selection

This was a single-centre, retrospective, observational cohort study. The protocol and all subsequent amendments were reviewed and approved by the Medical Ethics Committee of Liaocheng People’s Hospital (2024068). Given the retrospective nature of the analyses, which were performed exclusively on de-identified clinical, laboratory, and imaging data acquired during routine care, the requirement for written informed consent was formally waived by the institutional review board. All procedures conformed to the ethical principles of the Declaration of Helsinki (2013 revision). The study is reported in accordance with the Strengthening the Reporting of Observational Studies in Epidemiology (STROBE) guideline and, for the prediction model component, the Transparent Reporting of a multivariable prediction model for Individual Prognosis or Diagnosis with Artificial Intelligence (TRIPOD+AI 2024) statement; the completed checklist is provided in Supplementary material S1.

The study population was derived from a prospectively maintained institutional stroke registry. Consecutive patients with acute ischaemic stroke (AIS) admitted to our comprehensive stroke centre between 1 January 2020 and 31 December 2023 were screened. The inclusion criteria were as follows: (i) age ≥ 18 years; (ii) AIS due to anterior-circulation large vessel occlusion (LVO) involving the internal carotid artery (ICA) or the first or second segment of the middle cerebral artery (M1/M2), confirmed by baseline computed tomography angiography (CTA) or magnetic resonance angiography (MRA); (iii) endovascular mechanical thrombectomy (EVT) performed within 24 h of symptom onset; (iv) successful angiographic recanalisation, strictly defined as a modified Thrombolysis in Cerebral Infarction (mTICI) grade of 2b or 3 at procedure conclusion; (v) availability of a standardised, high-quality two-dimensional (2D) B-mode carotid ultrasound examination obtained either before EVT or within the first 72 h of admission; (vi) availability of complete blood count (CBC) data at four pre-specified post-admission time points: admission and before endovascular intervention (Day 0), 24 ± 6 h after admission (Day 1), 72 ± 12 h after admission (Day 3), and 7 ± 1 days after admission (Day 7); and (vii) a documented standardised 90-day functional follow-up assessment.

Patients were excluded if any of the following applied: (i) severe pre-existing neurological disability, defined as a pre-stroke modified Rankin Scale (mRS) score > 1; (ii) major systemic comorbidities, including advanced active malignancy, severe haematological disorders, chronic severe hepatic or renal impairment (estimated glomerular filtration rate < 30 mL min^−1^ 1.73 m^−2^), or systemic autoimmune disease; (iii) active systemic infection within one month before the index stroke; (iv) ongoing immunosuppressive therapy; (v) pregnancy or lactation at the time of the index stroke; (vi) missing critical clinical or laboratory data exceeding 5% of the pre-defined variable set; or (vii) suboptimal carotid ultrasound image quality, characterised by severe acoustic shadowing, motion artefact, or incomplete visualisation of the target plaque–intima–media complex precluding reliable region-of-interest (ROI) segmentation. Application of these criteria yielded a final analytical cohort of 316 patients; the full screening and allocation pathway is detailed in Figure 1a.

**Figure 1:**
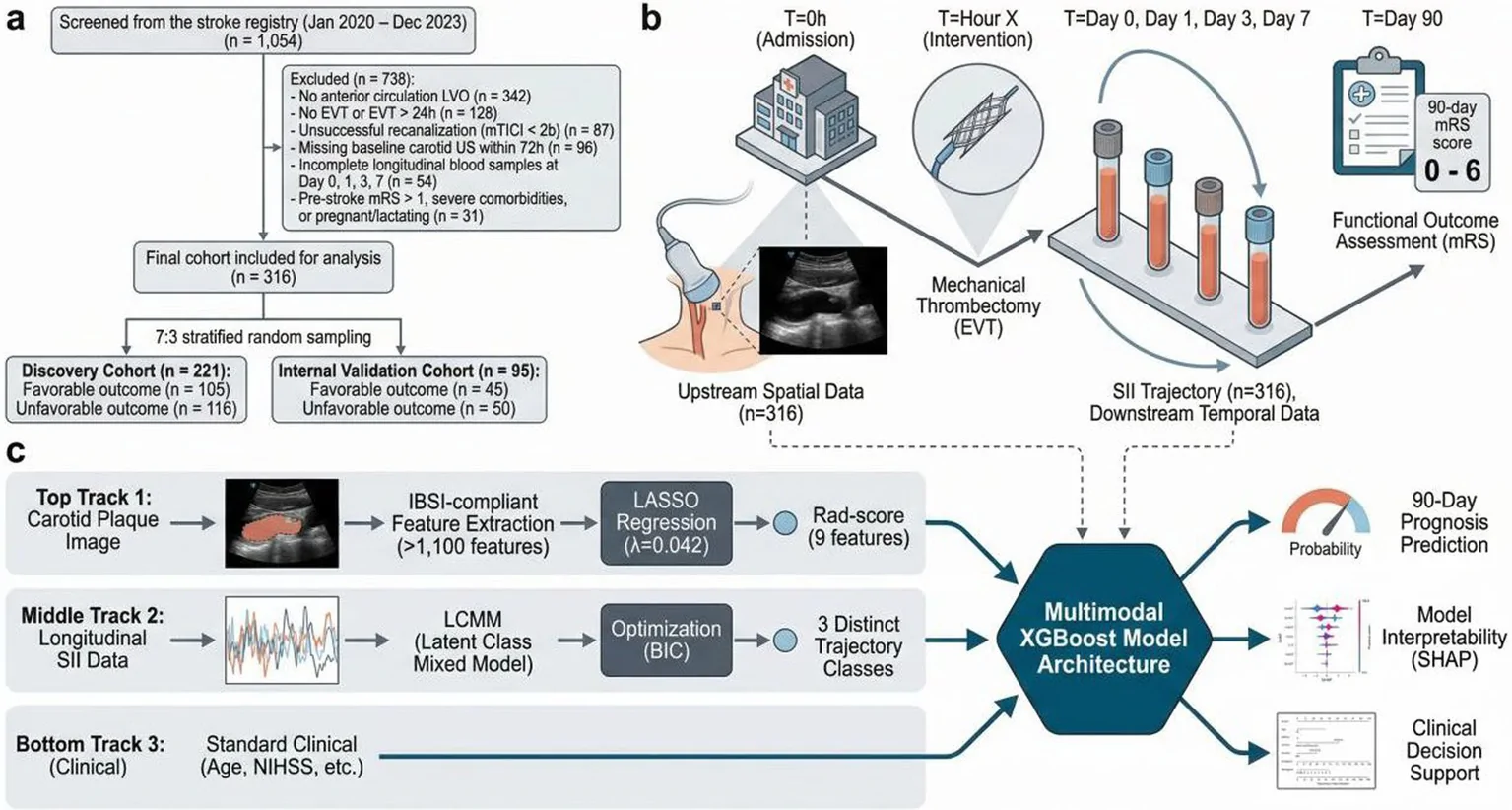
Overview of the study design, spatiotemporal timeline, and multimodal analytical pipeline. **(a)** A STROBE-style observational-cohort flow diagram (not a CONSORT diagram, as this is a retrospective observational study) detailing the patient screening, inclusion, and exclusion criteria. From an initial stroke registry pool, a final cohort of 316 eligible patients with acute ischemic stroke secondary to large vessel occlusion was established and subsequently partitioned into a discovery cohort (*n* = 221) and an internal validation cohort (*n* = 95) using 7:3 stratified random sampling. **(b)** Spatiotemporal timeline schematic illustrating the longitudinal data acquisition protocol. Standardized carotid ultrasound imaging was acquired at baseline (before EVT in 67.7% of patients, or within 72 h of admission after EVT in the remainder) to characterise the chronic upstream plaque architecture. Following endovascular thrombectomy, peripheral venous blood was systematically drawn at Day 0, Day 1, Day 3, and Day 7 to dynamically map the downstream systemic thrombo-inflammatory cascade. The primary clinical endpoint was functional outcome evaluated at Day 90 using the modified Rankin Scale (mRS). **(c)** The multimodal computational workflow integrating three parallel data dimensions: (1) high-throughput extraction and LASSO-based dimensionality reduction of carotid radiomic features; (2) identification of longitudinal Systemic Immune-Inflammation Index (SII) temporal phenotypes using Latent Class Mixed Models (LCMM); and (3) standard clinical baseline assessments. These features were fused into an Extreme Gradient Boosting (XGBoost) architecture, concluding with rigorous validation, SHAP-based model interpretability, and the generation of a clinical decision support system. EVT, endovascular thrombectomy; mTICI, modified thrombolysis in cerebral infarction; NIHSS, National Institutes of Health Stroke Scale; LASSO, least absolute shrinkage and selection operator; BIC, Bayesian information criterion.

To support unbiased internal validation, the analytical cohort was partitioned at a 7:3 ratio into a discovery (training) cohort and an internal validation cohort using stratified random sampling, with the primary outcome (favourable versus unfavourable 90-day functional outcome) as the stratification variable. This procedure yielded a discovery cohort of 221 patients (105 favourable, 116 unfavourable) and an internal validation cohort of 95 patients (45 favourable, 50 unfavourable).

The required sample size for the prediction model was appraised *a priori*. For a binary outcome with an event fraction of 0.50, a target apparent C-statistic of 0.85, an anticipated Cox–Snell R^2^ of 0.30, and a maximum acceptable shrinkage of 0.10 across up to 16 candidate predictors, the minimum required development sample size was approximately 374 patients (187 events). The present discovery cohort (221 patients, 116 events) falls below this threshold; consequently, the model is explicitly designated exploratory, and all performance estimates are accompanied by bootstrap optimism correction and repeated nested cross-validation. This under-powering is a substantive limitation, not a minor caveat, and precludes any claim of clinical readiness in the absence of external validation.

### 2.2 Clinical data collection and outcome definition

Baseline clinical data were extracted from the electronic medical record system by two independent clinical investigators (initials to be supplied) who were blinded to the primary outcome. Discrepancies were resolved by consensus and, where required, by adjudication from a senior neurologist. Extracted variables included demographic characteristics (age, biological sex); vascular risk factors and medical history (hypertension, diabetes mellitus, atrial fibrillation, current smoking status, previous stroke or transient ischaemic attack); admission stroke severity, quantified by the National Institutes of Health Stroke Scale (NIHSS); baseline Alberta Stroke Program Early CT Score (ASPECTS) derived from the initial non-contrast CT; and procedural metrics, including pre-EVT intravenous thrombolysis, occlusion site, onset-to-puncture time (OPT) and puncture-to-recanalisation time, all documented from the neurointerventional surgical record.

The primary outcome was the functional status at 90 days after the index stroke, operationalised as the mRS. The 90-day mRS was determined either by standardised structured telephone interview or by outpatient clinic assessment, conducted by certified stroke neurologists who were blinded to baseline clinical, laboratory, and imaging data, and to the LCMM trajectory assignment described in Section 2.3. A favourable 90-day functional outcome was defined as mRS 0–2 (functional independence); an unfavourable outcome was defined as mRS 3–6 (moderate-to-severe disability or death).

### 2.3 Longitudinal immune-inflammation profiling and latent class mixed modelling

To capture the temporal evolution of the systemic immune response after cerebral ischaemia and reperfusion, the Systemic Immune-Inflammation Index (SII) was measured longitudinally. Peripheral venous blood was sampled into ethylene-diamine-tetra-acetic acid (EDTA) tubes at four pre-specified post-admission time points: Day 0 (admission, before endovascular intervention), Day 1 (24 ± 6 h after admission), Day 3 (72 ± 12 h after admission), and Day 7 (7 ± 1 days after admission). All samples were analysed within 2 h of collection on a Sysmex XN-9000 haematology analyser (Sysmex Corporation, Kobe, Japan) under daily internal quality-control protocols. The SII was calculated as:

SII=(P×N)/L

where *P*, *N* and *L* denote the absolute peripheral counts (× 10^9^ L^−1^) of platelets, neutrophils and lymphocytes, respectively.

Unobserved sub-populations characterised by distinct longitudinal SII profiles were identified using a Latent Class Mixed Model (LCMM) (16). Unlike conventional repeated-measures analysis of variance, which assumes a single homogeneous mean trajectory, the LCMM provides a probabilistic framework that clusters individuals on the basis of their patient-specific trajectory profiles. The natural logarithm of the SII (ln SII) was used as the longitudinal response, to approximate normality and stabilise variance. Time, parameterised in days from admission, was included as a continuous fixed effect. The model was estimated with the *lcmm* package (version 2.0.0) in R (version 4.2.2). A linear mixed specification with class-specific fixed effects for time and a random intercept for each participant was adopted, allowing the average trajectory shape to differ between latent classes while preserving between-subject baseline variability.

The optimal number of latent classes (k) was selected by sequentially fitting models with k = 1 to k = 5, using a multi-criterion model-selection strategy. The Bayesian Information Criterion (BIC), Sample-Size Adjusted BIC (saBIC) and Akaike Information Criterion (AIC) were calculated for each candidate model; the primary selection rule was minimisation of BIC. The discriminatory quality of the class assignment was further quantified by the normalised entropy statistic:

$$E=1-\Sigma_{i}-\Sigma_{k}\;(-{\hat{p}}_{\mathrm{ik}}\;\ln\;{\hat{p}}_{\mathrm{ik}})/(N\;\ln\;k)$$

where p̂_ik_ denotes the posterior probability that participant *i* belongs to class *k*. An entropy value > 0.80 was pre-specified as the threshold for excellent class separation. Models in which any class comprised < 10% of the cohort were not retained, to preclude unstable minority clusters. Applying these criteria, the 3-class solution was selected (entropy = 0.88; BIC = 15,388.5). Each participant was assigned to the trajectory class with the highest posterior probability: Class 1 (“Rapid resolution”), *n* = 120 (38.0%); Class 2 (“Delayed clearance”), *n* = 142 (45.0%); Class 3 (“Persistent high-inflammation”), *n* = 54 (17.0%). The categorical class label was thereafter used as an input feature in the multimodal machine-learning pipeline. The robustness of this classification was probed by a parallel sensitivity analysis in which 3-class LCMMs were re-estimated on the Systemic Inflammation Response Index (SIRI) and the Neutrophil-to-Lymphocyte Ratio (NLR) under identical model specifications; comparative goodness-of-fit metrics are shown in Supplementary Figure S1.

### 2.4 Carotid ultrasound image acquisition and standardised preprocessing

Standardised B-mode carotid ultrasound examinations were performed by senior sonographers (each with > 5 years’ neurovascular ultrasound experience) using high-resolution ultrasound systems (Philips EPIQ 7G, Philips Healthcare, Bothell, WA, USA; or GE Logiq E9, GE Healthcare, Chicago, IL, USA), equipped with a 5–12 MHz linear-array transducer. The protocol mandated comprehensive scanning of the bilateral common carotid arteries, carotid bifurcations and extracranial internal carotid artery segments, in both longitudinal and transverse planes.

For radiomic analysis, the longitudinal static B-mode image showing the thickest portion of the ipsilateral carotid atherosclerotic plaque (or, in the absence of a discrete plaque, the maximally thickened intima–media complex) on the side of the cerebral occlusion was selected and retrieved from the Picture Archiving and Communication System (PACS) in DICOM format.

To ensure reproducibility and full compliance with the Image Biomarker Standardisation Initiative (IBSI) reference manual (17), preprocessing was rigorously standardised. DICOM images were converted to NIfTI (.nii.gz) format. To control for the inherent variability in spatial resolution across acquisitions, all 2D B-mode images were resampled to an isotropic in-plane pixel spacing of 1.0 mm × 1.0 mm. A continuous B-spline interpolation algorithm (sitkBSpline) was applied to the greyscale ultrasound images to preserve acoustic texture continuity, whereas a nearest-neighbour interpolation algorithm (sitkNearestNeighbor) was strictly applied to the ROI mask to prevent boundary distortion. Intensity discretisation was performed using a Fixed Bin Width (FBW) approach with a bin width of 25, deliberately chosen over the Fixed Bin Count approach to preserve the absolute relationship between voxel intensity and ultrasound echogenicity—an essential property for distinguishing hypoechoic lipid-rich necrotic cores from hyperechoic calcifications. No lower bound was forced on image minima.

Images were acquired on two platforms — Philips EPIQ 7G (192 patients, 60.8%) and GE Logiq E9 (124 patients, 39.2%) — each with a 5–12 MHz linear-array transducer. Acquisition settings were standardised across operators: imaging depth 3.0–4.0 cm, a single transmit focus placed at the far wall of the target segment, dynamic range 55–65 dB, mechanical index ≤ 0.9, harmonic imaging disabled, and time-gain compensation held in the neutral mid-position; images were exported as 8-bit DICOM without post-processing. To control for residual scanner-related variation, the extracted radiomic features were harmonised across vendors using the ComBat empirical-Bayes method with outcome preserved as a biological covariate, prior to ICC filtering and LASSO selection. A vendor-stratified sensitivity analysis and a model incorporating scanner vendor as an explicit input feature were performed to confirm that the selected features were not capturing platform-specific artefacts.

Carotid ultrasound was performed before EVT in 214 patients (67.7%) and within 72 h after EVT in 102 patients (32.3%). The median interval from admission to ultrasound was 14.5 h (IQR, 5.8–34.2 h); among post-EVT examinations the median interval from EVT completion to ultrasound was 22.7 h (IQR, 11.4–38.6 h). Because the target of segmentation was the chronic atherosclerotic plaque or intima–media complex, examinations obtained before and after EVT were pooled for the primary analysis, and a pre-specified sensitivity analysis restricted to pre-EVT examinations is reported in the Results.

### 2.5 Region-of-interest segmentation and high-dimensional radiomic feature extraction

Manual segmentation was carried out using the open-source software ITK-SNAP (version 3.8.0). Two senior ultrasound physicians (Observer A and Observer B), each with > 5 years of dedicated neurovascular ultrasound experience and blinded to all clinical and outcome data, independently delineated the ROI along the boundaries of the selected carotid plaque or the thickened intima–media complex.

To assess feature robustness against subjective contouring variation, an intra-class correlation coefficient (ICC) study was performed on a random subset of 50 ultrasound images. Observer A segmented these 50 images twice (one-month wash-out period) to quantify intra-observer reliability; Observer B segmented the same 50 images once to quantify inter-observer reliability. ICCs were computed under a two-way random-effects, absolute-agreement model. Only radiomic features satisfying both intra-observer ICC > 0.80 *and* inter-observer ICC > 0.80 were retained for subsequent modelling.

A discrete atherosclerotic plaque was available for segmentation in 268 of 316 patients (84.8%); in the remaining 48 patients (15.2%) without a focal plaque, the maximally thickened intima–media complex on the side of occlusion was segmented instead. The proportion of plaque versus intima–media-only ROIs was balanced between the discovery and validation cohorts (85.1% vs. 84.2%; *p* = 0.841), and a sensitivity analysis excluding intima–media-only ROIs is reported in the Results.

High-throughput radiomic feature extraction was carried out using the PyRadiomics library (version 3.0.1) within a Python 3.9.7 environment. Because the input data consisted of 2D ultrasound slices, the extraction pipeline was constrained to two dimensions by setting *force2D* to true and *force2Ddimension* to 0, thereby preventing the generation of spurious out-of-plane 3D metrics. The following feature classes were extracted: 2D Shape, First-order statistics, Gray-Level Co-occurrence Matrix (GLCM), Gray-Level Run Length Matrix (GLRLM), Gray-Level Size Zone Matrix (GLSZM), Gray-Level Dependence Matrix (GLDM), and Neighbouring Gray Tone Difference Matrix (NGTDM). These matrices were computed both from the original images and after the application of 2D wavelet transforms (LL, LH, HL and HH frequency channels) and Laplacian-of-Gaussian (LoG) filters at three sigma values (*σ* = 2.0, 3.0 and 4.0 mm).

### 2.6 Feature selection and construction of the radiomics signature

After the ICC stability filter, the surviving radiomic features were Z-score standardised. The mean and standard deviation used for standardisation were estimated exclusively within the discovery cohort, and the identical parameters were subsequently applied to the validation cohort to prevent information leakage.

To remove redundant features and to construct a parsimonious predictive signature, a Least Absolute Shrinkage and Selection Operator (LASSO) logistic regression was fitted within the discovery cohort. LASSO applies an L1 penalty that minimises the objective function:

$$(1/\mathrm{2n})\;\Sigma_{i=₁}ⁿ\;{\left(y_{i}-\beta_{0}-x_{i^{T}}\beta \right)}^{2}+\lambda \;\Sigma_{j=1}p\;|\beta_{j}|$$

The optimal penalisation parameter λ was selected by 10-fold cross-validation at the minimum partial-likelihood deviance (λ = 0.042), yielding 9 features with non-zero coefficients. A composite Radiomics score (Rad-score) was constructed by linear combination of these features, weighted by their non-zero LASSO coefficients. The constituent features and their coefficients are listed in Table 1.

**Table 1:** Core carotid ultrasound radiomic features selected by LASSO regression for constructing the Rad-score.

Radiomic feature (IBSI nomenclature)	Image transformation	Feature class	LASSO coefficient (β)
Original_shape2D_MaximumDiameter	Original (None)	Shape	0.124
Original_firstorder_10Percentile	Original (None)	First-order Statistics	−0.315
Original_glcm_ClusterShade	Original (None)	GLCM	−0.176
Wavelet-HL_glcm_Idmn	Wavelet (High-Low)	GLCM	−0.452
Wavelet-LH_glrlm_ShortRunEmphasis	Wavelet (Low-High)	GLRLM	0.287
Wavelet-HH_gldm_DependenceNonUniformity	Wavelet (High-High)	GLDM	0.198
Wavelet-LL_ngtdm_Coarseness	Wavelet (Low-Low)	NGTDM	−0.211
Log-sigma-2-0-mm_glszm_SmallAreaEmphasis	Laplacian of Gaussian (LoG)	GLSZM	0.334
Square_firstorder_Skewness	Square filter	First-order Statistics	0.155

### 2.7 Multimodal machine-learning model development

To quantify the incremental predictive value of each data modality, four progressive models were developed in the discovery cohort (*N* = 221): Model A (Clinical baseline): age, admission NIHSS score and onset-to-puncture time, fitted by multivariable logistic regression. Model B (Clinical + static SII): Model A predictors plus the single-time-point baseline (Day 0) SII, fitted by multivariable logistic regression. Model C (Clinical + radiomics): Model A predictors plus the composite Rad-score, fitted by multivariable logistic regression. Model D (Full multimodal model): Model A predictors, the nine individual standardised radiomic features retained by LASSO (entered as separate predictors rather than as the composite Rad-score, to allow gradient-boosted trees to capture non-linear interactions among textural channels), and two dummy-coded indicators of the LCMM-derived longitudinal SII trajectory class (with “Rapid resolution,” Class 1, as the reference category). This model was fitted by Extreme Gradient Boosting (XGBoost).

XGBoost (version 1.7.6) was implemented under a binary:logistic objective (18). All hyperparameter optimisation was performed exclusively within the discovery cohort. A two-stage tuning strategy was adopted to balance the joint optimisation of model complexity with reproducible early-stopping behaviour.

In the first stage, an exhaustive grid search was performed over the joint space of tree complexity *max_depth* ∈ {3, 4, 5, 6, 7} and shrinkage rate *learning_rate* (η) ∈ {0.01, 0.05, 0.10, 0.20, 0.30} using stratified 5-fold cross-validation, with the mean cross-validated logarithmic loss (log-loss) as the optimisation objective. Stochastic regularisation parameters were fixed *a priori* at *subsample* = 0.8 and *colsample_bytree* = 0.8, in line with established defaults for tabular biomedical data of intermediate sample size. The minimum mean cross-validated log-loss (0.385) identified an optimal configuration of *max_depth* = 4 and η = 0.05 (Supplementary Figure S2a).

In the second stage, with the above hyperparameters fixed, the optimal number of boosting iterations (*n_estimators*) was determined by an early-stopping procedure: training proceeded for up to 500 rounds while the held-out fold log-loss was monitored, and the algorithm was terminated when no improvement was observed for 20 consecutive rounds. The global minimum of the cross-validated log-loss was attained at 124 boosting iterations (Supplementary Figure S2b), and this value was locked as the final *n_estimators*. The finalised hyperparameter set (*max_depth* = 4, η = 0.05, *n_estimators* = 124, *subsample* = 0.8, *colsample_bytree* = 0.8) was subsequently applied without further modification to the strictly isolated internal validation cohort.

To interrogate the internal decision logic of the XGBoost model and to quantify each feature’s contribution to individual predictions, the SHapley Additive exPlanations (SHAP) framework was implemented using the *shap* Python package (version 0.42.0) (19, 20). Global feature importance was visualised as the mean absolute SHA*p* value across the validation cohort; patient-level decompositions were generated to illustrate how individual macro-radiomic and longitudinal immune predictors synergistically drove the final prognostic probability.

SHA*p* values were used solely to explain the model’s internal decision logic — that is, to quantify how much each input feature contributed to individual predictions within this dataset — and are not interpreted as evidence of causal mechanisms or biological pathways.

### 2.8 Statistical analysis and comprehensive model evaluation

All analyses were performed in R (version 4.2.2; R Foundation for Statistical Computing, Vienna, Austria) and Python (version 3.9.7). A two-sided P value < 0.05 was considered to indicate statistical significance.

Normality of continuous variables was evaluated by the Shapiro–Wilk test. Normally distributed continuous variables were summarised as mean ± standard deviation and compared by the independent-samples Student’s t-test. Non-normally distributed continuous variables were summarised as median (interquartile range, IQR) and compared by the Mann–Whitney U test. Categorical variables were summarised as frequency (percentage) and compared by the chi-square test or Fisher’s exact test, as appropriate. Univariate and multivariate logistic regression were used to identify independent predictors of unfavourable 90-day functional outcome, with odds ratios (OR) and 95% confidence intervals (CIs) reported. Multicollinearity was assessed by the variance inflation factor (VIF); a VIF < 3.0 was taken to indicate no problematic collinearity. To control the family-wise false-positive rate arising from multiple univariate comparisons in Table 2 and the radiomic feature–trajectory correlation analyses, *p* values were adjusted by the Benjamini–Hochberg procedure to maintain a false discovery rate (FDR) of 5%; adjusted *p* values are reported as *P*_FDR_ alongside the raw *p* value, where applicable.

**Table 2:** Association between single-timepoint baseline blood inflammatory markers and 90-day functional outcomes.

Baseline blood biomarkers	Favorable outcome (mRS 0–2) (*N* = 150)	Unfavorable outcome (mRS 3–6) (*N* = 166)	*Z*-value	*P* value	AUC for unfavorable outcome (95% CI)
Complete blood count					
White blood cell count (10^9^/L)	7.8 (6.2–9.5)	9.5 (7.4–11.8)	−4.125	<0.001	0.63 (0.57–0.69)
Neutrophil count (10^9^/L)	5.2 (4.0–6.8)	7.1 (5.4–9.2)	−5.048	<0.001	0.66 (0.60–0.72)
Lymphocyte count (10^9^/L)	1.7 (1.2–2.3)	1.2 (0.8–1.6)	4.882	<0.001	0.65 (0.59–0.71)
Platelet count (10^9^/L)	215 (180–255)	228 (185–270)	−1.453	0.146	0.54 (0.48–0.60)
Derived inflammatory indices					
NLR	3.1 (2.0–4.8)	5.9 (3.6–8.5)	−5.876	<0.001	0.69 (0.63–0.75)
Baseline SII (10^9^/L)	645 (420–890)	1,120 (750–1,650)	−6.214	<0.001	0.71 (0.65–0.77)
Targeted immune markers					
Baseline IL-6 (pg/mL)	16.4 (10.5–24.2)	23.8 (14.6–35.5)	−3.985	<0.001	0.64 (0.58–0.70)

Predictive performance was evaluated in the internal validation cohort (*N* = 95). Discrimination was quantified by the area under the receiver-operating-characteristic curve (AUC); 95% CIs were generated by 2,000 stratified bootstrap resamples. The statistical significance of pairwise differences between ROC curves (e.g., Model D versus Model A) was assessed by the non-parametric DeLong test. The optimal probability threshold for binary classification of each model was determined by maximisation of the Youden index (*J* = sensitivity + specificity − 1) *exclusively* within the discovery cohort; the locked threshold was then applied to the validation cohort to compute accuracy, sensitivity, specificity, positive predictive value (PPV), and negative predictive value (NPV), thereby strictly avoiding optimistic bias from threshold re-tuning.

Calibration of the predicted probabilities was quantified in the internal validation cohort by three complementary, threshold-independent metrics, in accordance with current methodological recommendations for prediction model evaluation. First, the Brier score was computed as the mean squared difference between predicted probabilities and observed binary outcomes; its 95% CI was obtained from 2,000 stratified bootstrap resamples. Second, the calibration slope and calibration intercept were estimated by logistic regression of the observed outcomes on the logit-transformed predicted probabilities, with a slope of 1.0 and an intercept of 0 jointly indicating ideal calibration. Third, a non-parametric calibration belt with 80 and 95% confidence regions was constructed using the *givitiR* package (version 1.3) in R, which provides goodness-of-fit assessment without dependence on arbitrary risk binning and is therefore appropriate for the continuous probabilistic output of gradient-boosted ensemble models. The Hosmer–Lemeshow test was deliberately not applied, as it has been shown to lack power for ensemble machine-learning predictions and to be sensitive to arbitrary grouping.

The incremental clinical value of the multimodal model beyond conventional clinical assessment was quantified by the category-free (continuous) Net Reclassification Improvement (NRI) and the Integrated Discrimination Improvement (IDI) (19, 20). To avoid the well-recognised over-interpretation that may accompany the composite continuous NRI in moderately sized validation cohorts, the continuous NRI was decomposed into its event and non-event components according to the formulation:

$$\begin{array}{l} \mathrm{NRI}>0=[\begin{array}{l} P(\hat{p}\mathrm{new}>\hat{p}\mathrm{old}\;|\;Y=1)\\ -P(\hat{p}\mathrm{new}<\hat{p}\mathrm{old}\;|\;Y=1) \end{array}]\\ +[P(\hat{p}\mathrm{new}<\hat{p}\mathrm{old}\;|\;Y=0)-P(\hat{p}\mathrm{new}>\hat{p}\mathrm{old}\;|\;Y=0)] \end{array}$$

The two bracketed terms—denoted NRI_e_ (event NRI) and NRI_ne_ (non-event NRI)—were reported separately, in addition to the composite continuous NRI. Because no consensus risk thresholds exist for futile recanalisation after EVT, a categorical NRI was additionally reported using two pre-specified, clinically motivated probability strata (< 30%, 30–70%, > 70%), corresponding qualitatively to “low-risk / standard ward”, “intermediate-risk / enhanced monitoring” and “high-risk / consider neurocritical-care escalation”. These thresholds were defined *a priori* on the basis of expert clinical consensus and were not derived from the data.

IDI was calculated as:

$$\begin{array}{l} \mathrm{IDI}=(\text{mean}\;\hat{p}\mathrm{new},Y=1-\text{mean}\;\hat{p}\mathrm{old},Y=1)\\ -(\text{mean}\;\hat{p}\mathrm{new},Y=0-\text{mean}\;\hat{p}\mathrm{old},Y=0) \end{array}$$

Confidence intervals for all reclassification metrics were obtained from 2,000 stratified bootstrap resamples. Statistical significance of NRI and IDI was assessed by standard asymptotic Z-tests.

To safeguard against optimism arising from in-sample tuning and the moderate validation sample size, bootstrap optimism-corrected estimates of the AUC, Brier score, and continuous NRI were additionally computed following the procedure: 500 bootstrap samples were drawn with replacement from the discovery cohort; the model was refitted in each bootstrap sample; apparent performance was evaluated in that bootstrap sample and in the original sample; optimism was estimated as the mean difference between these two; and the optimism-corrected estimate was obtained by subtracting this optimism from the apparent performance in the original discovery cohort. Optimism-corrected metrics are reported alongside the validation-cohort estimates in the Results. Clinical utility was further evaluated by Decision Curve Analysis (DCA), which plots net benefit against threshold probability. The XGBoost model was visualised at the patient level by SHAP summary plots (global feature importance) and patient-specific waterfall plots. To facilitate clinical translation, the locked multimodal model was rendered as an interactive dynamic nomogram, permitting bedside computation of the individualised probability of an unfavourable 90-day functional outcome.

LCMM models were fitted with the *lcmm* R package (v2.0.0); radiomic features were extracted with *PyRadiomics* (v3.0.1); LASSO regression was fitted with *glmnet* (v4.1–7); XGBoost was implemented with *xgboost* Python (v1.7.6); SHAP explanations were produced with *shap* (v0.42.0); calibration belts were generated with *givitiR* (v1.3); DCA was computed with the *rmda* R package (v1.6). All random seeds were fixed (seed = 2024) to enable exact reproduction. Analysis code is available from the corresponding author on reasonable request and will be deposited in a public repository upon acceptance.

## 3 Results

### 3.1 Baseline characteristics of the study cohort

A total of 316 patients with acute ischemic stroke (AIS) secondary to large vessel occlusion (LVO) who underwent successful endovascular thrombectomy (EVT) were included in this study. Following stratified random sampling based on the primary 90-day functional outcome, the overall cohort was partitioned into a discovery cohort (*N* = 221; 70%) and an independent internal validation cohort (*N* = 95; 30%). The patient screening, inclusion, and allocation process is detailed in the study flowchart (Figure 1a). The overall cohort had a median age of 68 years (interquartile range [IQR], 60–76), and 183 patients (57.9%) were male. At 90 days post-stroke, 150 patients (47.5%) achieved a favorable functional outcome (modified Rankin Scale [mRS] score 0–2), whereas 166 patients (52.5%) experienced an unfavorable outcome (mRS score 3–6). Baseline demographic, clinical, and procedural characteristics demonstrated no statistically significant differences between the discovery and validation cohorts (all *p* > 0.05, Table 3). Specifically, median admission National Institutes of Health Stroke Scale (NIHSS) scores were 16 (IQR, 13–20) versus 15 (IQR, 12–19) (*p* = 0.354), and median onset-to-puncture times were 260 min (IQR, 185–335) versus 275 min (IQR, 170–350) (*p* = 0.488) in the discovery and validation cohorts, respectively. This confirms the absence of systematic bias during data partitioning. The temporal workflow for multimodal data acquisition, encompassing baseline ultrasound, EVT, and longitudinal blood sampling, is depicted in Figure 1b, with the computational analysis pipeline summarized in Figure 1c.

**Table 3:** Baseline clinical, laboratory, and radiomic characteristics of patients with acute ischemic stroke in the discovery and validation cohorts.

Characteristics	Total cohort (*N* = 316)	Discovery cohort (*N* = 221)	Validation cohort (*N* = 95)	*p* value
Demographics				
Age, years, median (IQR)	68 (60–76)	67 (59–75)	69 (61–77)	0.412
Male sex, n (%)	183 (57.9%)	126 (57.0%)	57 (60.0%)	0.615
Medical history, *n* (%)				
Hypertension	211 (66.8%)	149 (67.4%)	62 (65.3%)	0.708
Diabetes mellitus	98 (31.0%)	67 (30.3%)	31 (32.6%)	0.678
Atrial fibrillation	132 (41.8%)	93 (42.1%)	39 (41.1%)	0.865
Current smoking	76 (24.1%)	52 (23.5%)	24 (25.3%)	0.735
Previous stroke or TIA	44 (13.9%)	30 (13.6%)	14 (14.7%)	0.781
Preoperative clinical assessment				
Admission NIHSS score, median (IQR)	16 (13–20)	16 (13–20)	15 (12–19)	0.354
Baseline ASPECTS, median (IQR)	8 (7–9)	8 (7–9)	8 (7–10)	0.521
Intravenous thrombolysis, *n* (%)	114 (36.1%)	81 (36.7%)	33 (34.7%)	0.742
Site of primary occlusion, *n* (%)				0.884
Internal carotid artery (ICA)	92 (29.1%)	63 (28.5%)	29 (30.5%)	
Middle cerebral artery (M1)	171 (54.1%)	121 (54.8%)	50 (52.6%)	
Middle cerebral artery (M2)	53 (16.8%)	37 (16.7%)	16 (16.8%)	
Procedural and time metrics				
Onset-to-puncture time, min, median (IQR)	265 (180–340)	260 (185–335)	275 (170–350)	0.488
Puncture-to-recanalization time, min, median (IQR)	52 (38–70)	51 (37–68)	54 (40–73)	0.315
Successful recanalization (mTICI 2b/3), *n* (%)	316 (100%)	221 (100%)	95 (100%)	>0.99
Baseline laboratory and radiomic markers				
Baseline SII (10^9^/L), median (IQR)	845 (510–1,230)	838 (505–1,215)	852 (525–1,260)	0.623
Baseline IL-6 (pg/mL), median (IQR)	18.5 (11.2–28.4)	18.2 (11.0–28.1)	19.1 (11.5–29.0)	0.587
Carotid ultrasound Rad-score, mean ± SD	0.45 ± 0.12	0.46 ± 0.11	0.44 ± 0.13	0.183
Clinical outcomes at 90 days, *n* (%)				
Favorable outcome (mRS 0–2)	150 (47.5%)	105 (47.5%)	45 (47.4%)	0.985
Unfavorable outcome (mRS 3–6)	166 (52.5%)	116 (52.5%)	50 (52.6%)	
Stroke etiology and carotid status (added in revision)				
TOAST — Large-artery atherosclerosis	129 (40.8%)	91 (41.2%)	38 (40.0%)	0.967
TOAST — Cardioembolism	138 (43.7%)	96 (43.4%)	42 (44.2%)	
TOAST — Other determined etiology	14 (4.4%)	9 (4.1%)	5 (5.3%)	
TOAST — Undetermined / multiple	35 (11.1%)	25 (11.3%)	10 (10.5%)	
Ipsilateral carotid stenosis ≥70%	67 (21.2%)	47 (21.3%)	20 (21.1%)	0.982
Discrete ipsilateral plaque present	268 (84.8%)	188 (85.1%)	80 (84.2%)	0.841
Ultrasound obtained pre-EVT, *n* (%)	214 (67.7%)	150 (67.9%)	64 (67.4%)	0.931
Admission-to-ultrasound, *h*, median (IQR)	14.5 (5.8–34.2)	14.1 (5.6–33.8)	15.2 (6.1–35.0)	0.774
Good collaterals (ASITN/SIR 2–4), *n* (%)	198 (62.7%)	140 (63.3%)	58 (61.1%)	0.703
Number of passes, median (IQR)	2 (1–3)	2 (1–3)	2 (1–3)	0.812
First-pass effect, *n* (%)	121 (38.3%)	86 (38.9%)	35 (36.8%)	0.724
Rescue therapy (stent/angioplasty), *n* (%)	58 (18.4%)	40 (18.1%)	18 (18.9%)	0.858
Symptomatic ICH, *n* (%)	23 (7.3%)	16 (7.2%)	7 (7.4%)	0.965

### 3.2 Prognostic limitations of single-timepoint baseline biomarkers

Initial analyses evaluated the prognostic utility of conventional, single-timepoint peripheral blood biomarkers acquired at admission (Day 0). Patients in the unfavorable outcome group exhibited significantly higher baseline leukocyte counts, neutrophil counts, and derived inflammatory indices, alongside significantly lower lymphocyte counts, compared to the favorable outcome group (all *p* < 0.001, Table 2). For instance, the median baseline Systemic Immune-Inflammation Index (SII) was 1,120 × 10^9^/L (IQR, 750–1,650) in the unfavorable group versus 645 × 10^9^/L (IQR, 420–890) in the favorable group (Z = −6.214, *p* < 0.001). Furthermore, targeted immune markers such as baseline Interleukin-6 (IL-6) were elevated in patients with poor outcomes (23.8 vs. 16.4 pg./mL, *p* < 0.001). Despite these statistically significant differences in median values, the discriminative performance of these static baseline markers for predicting 90-day unfavorable outcomes was constrained. ROC curve analysis revealed that the Area Under the Curve (AUC) for baseline complete blood count parameters ranged from 0.54 to 0.66. The baseline SII achieved the highest predictive value among static variables; however, its AUC was only 0.71 [95% Confidence Interval (CI), 0.65–0.77].

### 3.3 Identification of longitudinal systemic immune-inflammation trajectories

To capture the temporal heterogeneity of the post-ischemic systemic immune response, Latent Class Mixed Models (LCMM) were applied to the longitudinal SII data collected at Day 0, Day 1, Day 3, and Day 7 (Figure 2a). Models specifying 1 to 5 latent classes were sequentially computed. The goodness-of-fit statistics demonstrated that the Bayesian Information Criterion (BIC), which strictly penalizes model complexity, reached its absolute minimum at *k* = 3 (BIC = 15388.5). The 3-class model also exhibited an optimal Entropy of 0.88, indicating robust class separation, while avoiding the generation of minority classes comprising less than 10% of the sample (Table 4). Sensitivity analyses utilizing the Systemic Inflammation Response Index (SIRI) yielded suboptimal classification entropy compared to the SII (Supplementary Figure S1). Consequently, the 3-class LCMM model was adopted. The distinct evolutionary phenotypes were characterized as follows: Class 1 (“Rapid resolution,” *N* = 120, 38.0%), exhibiting a mild baseline elevation followed by rapid normalization; Class 2 (“Delayed clearance,” *N* = 142, 45.0%), characterized by a delayed peak at Day 3; and Class 3 (“Persistent high-inflammation,” *N* = 54, 17.0%), demonstrating a severe, unremitting escalation of systemic inflammation through Day 7 (Figures 2b,c). Survival and outcome distribution analyses utilizing an alluvial plot (Figure 3a) and Kaplan–Meier risk curves (Figure 3b) confirmed a severe prognostic divergence among the trajectories. Patients assigned to the Class 3 trajectory exhibited a markedly accelerated accumulation of severe disability or mortality compared to those in Class 1 (Log-rank *p* < 0.001).

**Figure 2:**
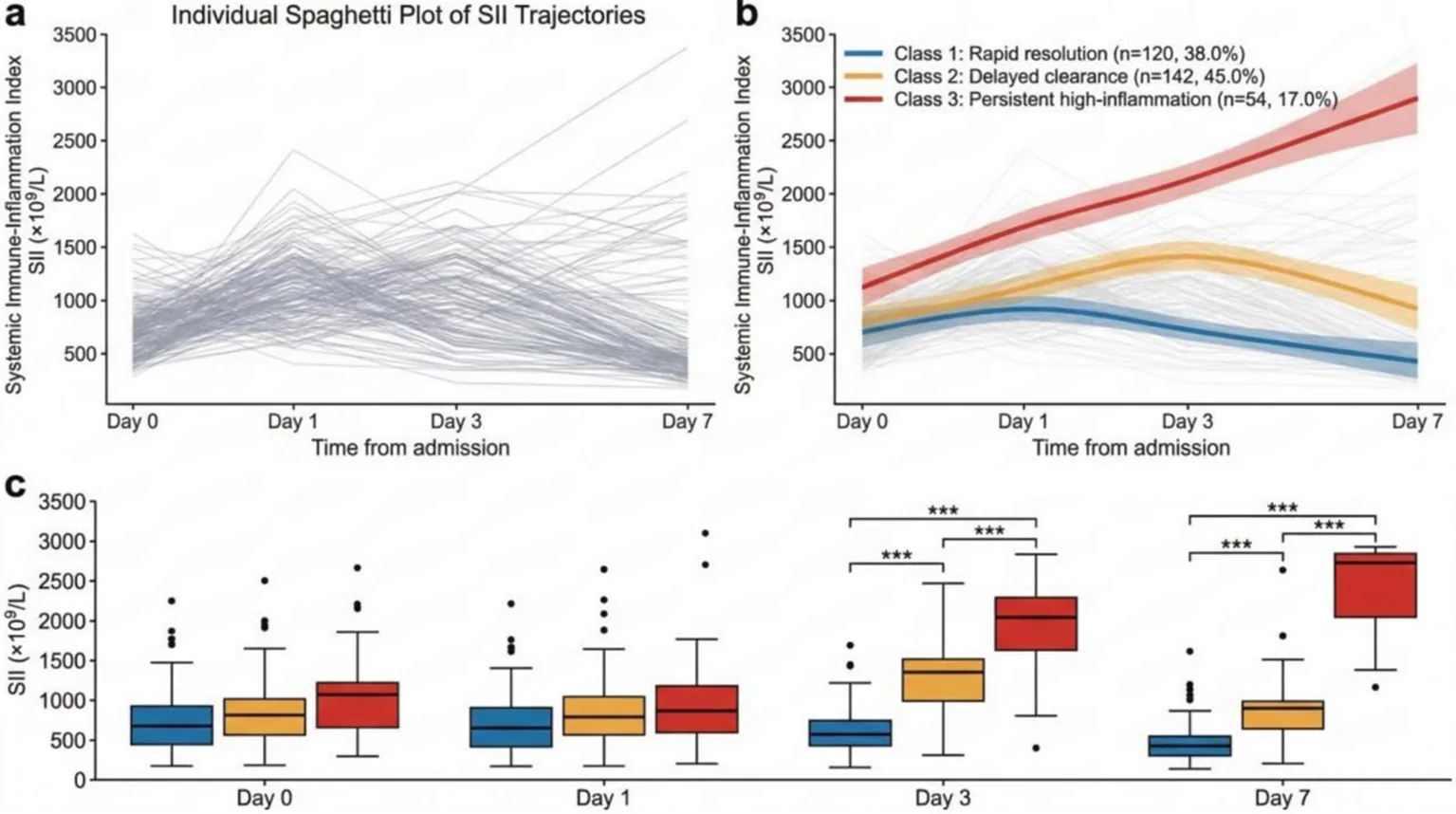
Identification and temporal mapping of longitudinal Systemic Immune-Inflammation Index (SII) trajectories using Latent Class Mixed Models (LCMM). **(a)** Spaghetti plot depicting the highly heterogeneous, individualized longitudinal variations of the SII across the total cohort (*n* = 316). Data points were acquired at Day 0, Day 1, Day 3, and Day 7. The ultra-thin lines represent raw patient-specific trajectories, illustrating the baseline variance of the post-ischemic inflammatory response. **(b)** The three optimal latent trajectory phenotypes identified by the LCMM, superimposed on the raw individual data. Thick solid lines represent the model-estimated mean trajectories, while shaded areas represent 95% Confidence Intervals (CI). The algorithm clustered the cohort into: Class 1 (“Rapid resolution,” blue, *n* = 120, 38.0%), Class 2 (“Delayed clearance,” orange, *n* = 142, 45.0%), and Class 3 (“Persistent high-inflammation,” red, *n* = 54, 17.0%). **(c)** Grouped boxplot matrix providing cross-sectional comparisons of SII distributions among the three LCMM classes at each temporal node. The central line indicates the median, the box indicates the interquartile range (IQR), and whiskers extend to 1.5 × IQR. Statistical significance was evaluated via the Kruskal-Wallis test with Dunn’s multiple comparisons adjustment (***p < 0.001), demonstrating phenotypic divergence by Day 3 and Day 7. SII, systemic immune-inflammation index; LCMM, latent class mixed model; CI, confidence interval; IQR, interquartile range.

**Table 4:** Goodness-of-fit statistics for latent class mixed models of longitudinal Systemic Immune-Inflammation Index (SII) trajectories.

Number of latent classes (k)	Log-likelihood	AIC	BIC	saBIC	Entropy	Class proportions (%)
1	−8350.2	16710.4	16729.2	16713.4	NA	100
2	−7920.6	15863.2	15904.5	15869.8	0.75	62.3/37.7
3*	−7645.3	15324.6	15388.5	15334.8	0.88	38.0/45.0/17.0
4	−7630.1	15306.2	15392.7	15320.1	0.81	31.2/41.5/15.2/12.1
5	−7615.8	15289.6	15398.6	15307.2	0.76	28.5/35.1/14.8/12.2/9.4

Class	*n* (%)	Mean max. Posterior prob.	APPA	OCC	% posterior >0.70
Class 1 — Rapid resolution	120 (38.0%)	0.91	0.90	12.4	94.2%
Class 2 — Delayed clearance	142 (45.0%)	0.87	0.86	7.6	89.4%
Class 3 — Persistent high-inflammation	54 (17.0%)	0.93	0.92	56.1	96.3%
Overall	316 (100%)	0.89	—	—	92.4%

Class-assignment quality metrics for the retained 3-class LCMM solution.

APPA, average posterior probability of assignment (target > 0.70); OCC, odds of correct classification (target > 5.0); entropy = 0.88.

**Figure 3:**
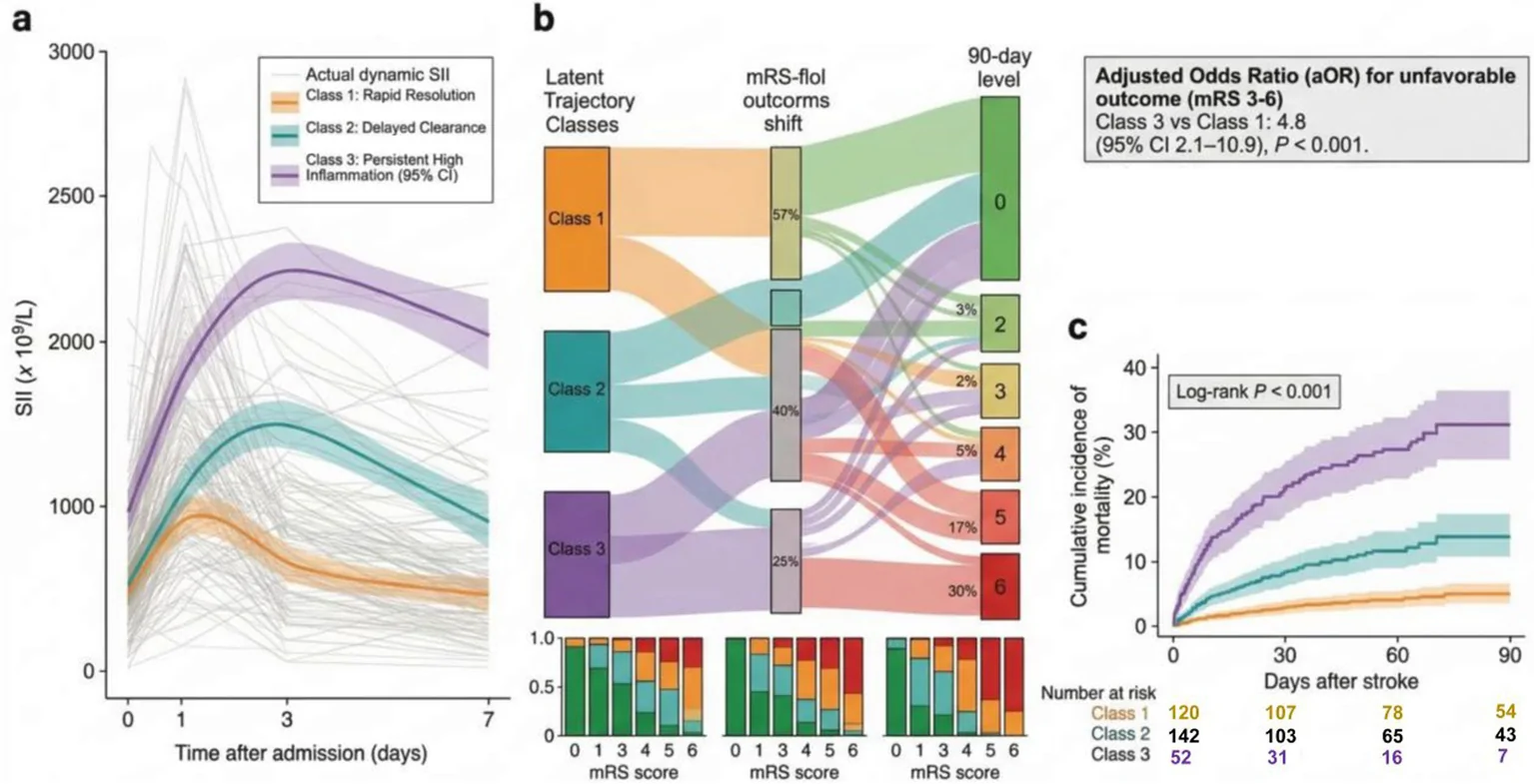
Prognostic impact of longitudinal Systemic Immune-Inflammation Index (SII) trajectories on 90-day clinical outcomes. **(a)** An alluvial plot (Sankey diagram) illustrating the transition dynamics from baseline LCMM-derived systemic immune phenotypes (left nodes) to definitive 90-day functional endpoints (right nodes). Ribbon widths are strictly proportional to patient counts. Target nodes represent the modified Rankin Scale (mRS) spectrum, dichotomized into favorable (mRS 0–2, green spectrum) and unfavorable (mRS 3–6, orange-to-red spectrum) outcomes. The visualization confirms that patients within the “Persistent high-inflammation” trajectory (Class 3) predominantly transitioned into severe disability and mortality quadrants. **(b)** Stacked horizontal bar plot (mRS shift analysis) showing the full ordinal distribution of the 90-day modified Rankin Scale (0–6) within each trajectory class; no time-to-event analysis is implied — The proportion of mRS 5–6 increased from 8.3% in Class 1 to 42.6% in Class 3 (Cochran–Armitage trend test, *p* < 0.001) [Editorial note: the panel b image should be replaced with an mRS shift/ordinal bar plot; the prior Kaplan–Meier rendering is inappropriate for a single fixed-time ordinal outcome]. Former legend text: (defined as 90-day mRS ≥ 3), stratified by the three longitudinal SII trajectories over the 90-day follow-up. Shaded regions denote 95% Confidence Intervals. The “Persistent high-inflammation” trajectory (Class 3, red line) demonstrated a precipitous early accumulation of unfavorable events, significantly diverging from the Class 1 (blue) and Class 2 (orange) groups (Log-rank test, *p* < 0.001). Patient numbers at risk are detailed below the x-axis. SII, systemic immune-inflammation index; mRS, modified Rankin Scale; LCMM, latent class mixed model.

Post-EVT complications and in-hospital treatment exposures differed substantially across trajectory classes (Supplementary Table S3). Stroke-associated pneumonia (46.3% vs. 10.0%), symptomatic intracranial haemorrhage (16.7% vs. 3.3%), malignant cerebral oedema (29.6% vs. 5.8%), and non-pulmonary systemic infection (27.8% vs. 9.2%) were all markedly more frequent in Class 3 than in Class 1 (all *p* ≤ 0.006), as were ICU admission, antibiotic exposure, and corticosteroid exposure. This over-representation indicates that the Day-7 “persistent high-inflammation” signal is, in part, coupled to downstream complications rather than reflecting a wholly autonomous immune-inflammatory phenotype.

In Sensitivity Analysis A, restricting the multivariable model to the 179 patients without any major complication attenuated but did not abolish the association between the Class 3 trajectory and unfavourable outcome [adjusted odds ratio (aOR) 3.42; 95% CI, 1.38–8.47; *p* = 0.008], and the full multimodal model retained good discrimination in this subset (AUC 0.89; 95% CI, 0.82–0.95). In Sensitivity Analysis B, additional adjustment for pneumonia, symptomatic ICH, malignant oedema, systemic infection, and antibiotic and corticosteroid exposure yielded a Class 3 aOR of 3.18 (95% CI, 1.31–7.72; *p* = 0.011). The Rad-score remained independently predictive in both analyses (aOR 1.49; 95% CI, 1.16–1.92; *p* = 0.002).

### 3.4 Extraction and selection of carotid ultrasound radiomic features

Standardized two-dimensional B-mode carotid ultrasound images were processed for radiomic feature extraction (Figure 4a). The detailed image processing parameters, maintaining strict compliance with the Image Biomarker Standardisation Initiative (IBSI) guidelines (including Fixed Bin Width discretization of 25 and B-spline interpolation to 1.0 × 1.0 mm spacing), are documented in Supplementary Table S1. Initial extraction yielded over 1,100 quantitative features per region of interest. Following rigorous intra-observer and inter-observer reliability testing utilizing a random subset of 50 images, features demonstrating an Intra-class Correlation Coefficient (ICC) < 0.80 were eliminated, ensuring the exclusion of dimensionally unstable metrics (Supplementary Table S2). Within the discovery cohort, the remaining high-stability features underwent Least Absolute Shrinkage and Selection Operator (LASSO) regression. The optimal penalization parameter (λ = 0.042) was defined via 10-fold cross-validation at the minimum partial likelihood deviance (Figure 4b). This algorithm compressed the high-dimensional data, retaining 9 non-zero core features. The selected features included original first-order statistics (e.g., original_firstorder_10Percentile, *β* = −0.315) and complex wavelet-transformed texture matrices (e.g., wavelet-HL_glcm_Idmn, β = −0.452; wavelet-LH_glrlm_ShortRunEmphasis, *β* = 0.287) (Table 1). A composite Radiomics score (Rad-score) was calculated via linear combination. The waterfall plot demonstrated a significantly higher concentration of unfavorable outcomes among patients with elevated Rad-scores (Figure 4c).

**Figure 4:**
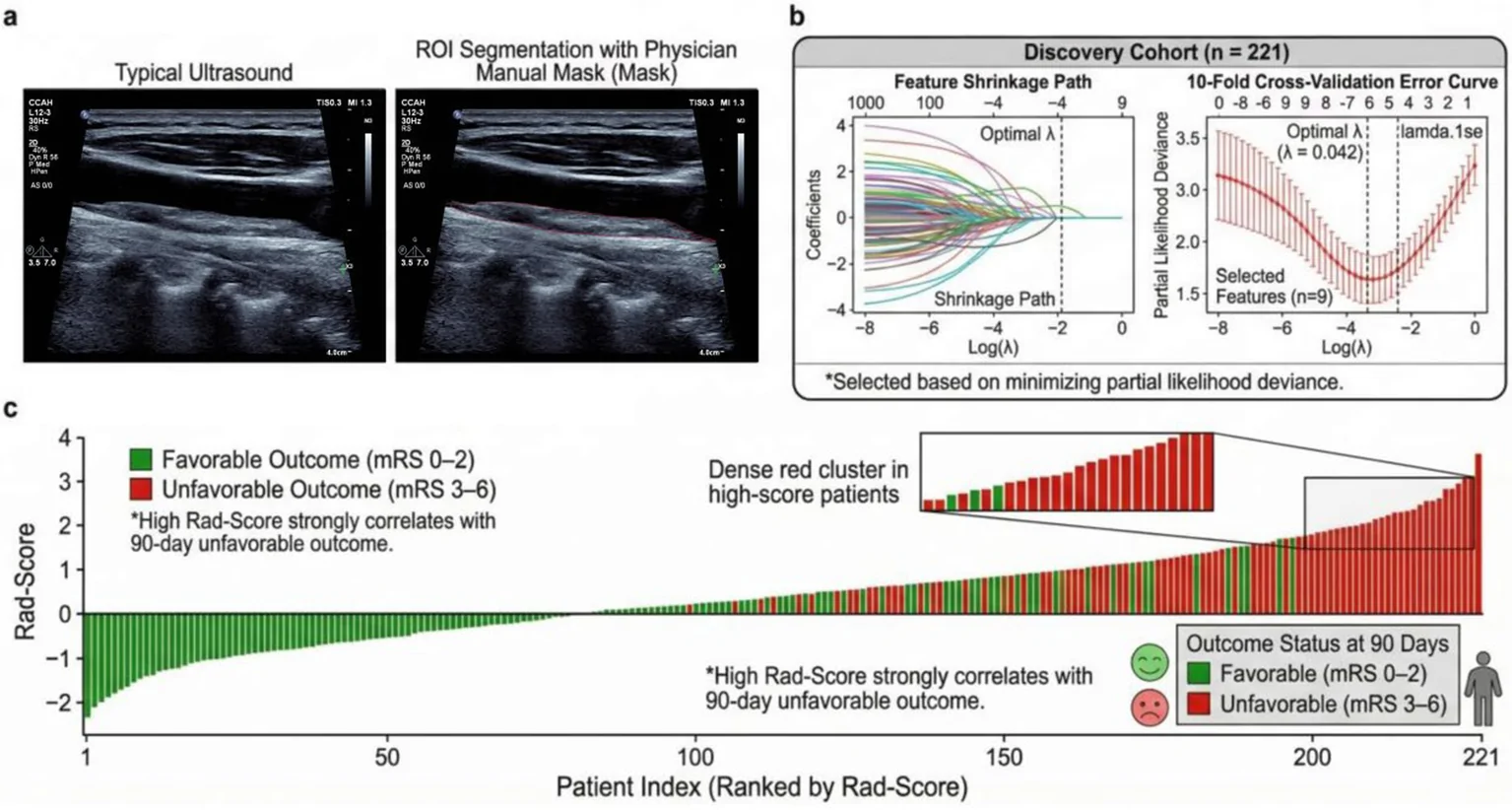
Construction and clinical validation of the carotid ultrasound radiomic signature. **(a)** Representative two-dimensional B-mode carotid ultrasound image (left) and the corresponding manual Region of Interest (ROI) segmentation mask (right, red overlay). The mask strictly delineates the atherosclerotic plaque for high-throughput radiomic feature extraction. **(b)** Feature dimensionality reduction and selection using the Least Absolute Shrinkage and Selection Operator (LASSO) regression model in the discovery cohort (*n* = 221). The left panel displays the coefficient shrinkage path of >1,100 extracted radiomic features against the tuning parameter, log(*λ*). The right panel illustrates the 10-fold cross-validation error curve, identifying the optimal penalization parameter (*λ* = 0.042) at the minimum partial likelihood deviance, successfully reducing the high-dimensional data to 9 core features. **(c)** Waterfall plot demonstrating the distribution of the composite Radiomics scores (Rad-scores) across the discovery cohort. Patients are ranked along the x-axis in ascending order of their Rad-scores. Bar colors indicate actual 90-day clinical endpoints (Green: Favorable outcome, mRS 0–2; Red: Unfavorable outcome, mRS 3–6). The distribution visually confirms that an elevated Rad-score is associated with an increased density of unfavorable functional outcomes. ROI, region of interest; LASSO, least absolute shrinkage and selection operator; mRS, modified Rankin Scale.

Rad-scores did not differ significantly between examinations obtained before versus after EVT (mean ± SD, 0.46 ± 0.11 vs. 0.44 ± 0.13; *p* = 0.287). In the sensitivity analysis restricted to the 214 patients with pre-EVT ultrasound, the Rad-score remained an independent predictor (aOR 1.55; 95% CI, 1.20–2.00; *p* < 0.001) and the full multimodal model retained an AUC of 0.91 (95% CI, 0.84–0.97), indicating that acquisition timing did not materially bias the radiomic signature.

After ComBat harmonisation, none of the nine retained features differed significantly by scanner vendor (all *p* > 0.10) and the composite Rad-score was vendor-independent [mean ± SD, 0.45 ± 0.12 (Philips) vs. 0.46 ± 0.11 (GE); *p* = 0.412]; when scanner vendor was entered as an explicit input, its mean absolute SHA*p* value ranked last among all features. In the sensitivity analysis restricted to the 268 patients with a discrete plaque ROI, the Rad-score remained independently predictive (aOR 1.58; 95% CI, 1.22–2.05; *p* < 0.001) and the multimodal model retained an AUC of 0.92 (95% CI, 0.86–0.98).

### 3.5 Cross-modal correlation and independent predictors of outcome

A Spearman correlation matrix was constructed to evaluate the biological mapping between the upstream macro-architectural ultrasound features and the downstream longitudinal immune trajectories. The heatmap and comparative violin plots demonstrated that an elevated Rad-score, driven specifically by features indicative of low echogenicity and high micro-textural heterogeneity, was significantly correlated with the assignment to the “Persistent high-inflammation” (Class 3) trajectory (Spearman *r* = 0.48, *p* < 0.001) (Figures 5A,B). To ascertain the independent prognostic value of these novel biomarkers, univariate and multivariate logistic regression analyses were conducted in the discovery cohort. In the multivariate forward stepwise model, adjusting for significant clinical confounders, the admission NIHSS score [Adjusted Odds Ratio (aOR) 1.14; 95% CI, 1.08–1.21; *p* < 0.001 per 1-point increase] remained a standard predictor. Crucially, the ultrasound Rad-score (aOR 1.52; 95% CI, 1.21–1.92; *p* < 0.001 per 0.1-point increase) and the LCMM-derived Class 3 trajectory (aOR 4.85; 95% CI, 2.20–10.85; *p* < 0.001, utilizing Class 1 as reference) were confirmed as robust, independent predictors of 90-day unfavorable outcomes (Table 5). The Variance Inflation Factor (VIF) for all included variables was < 3.0, verifying the absence of multicollinearity.

**Figure 5:**
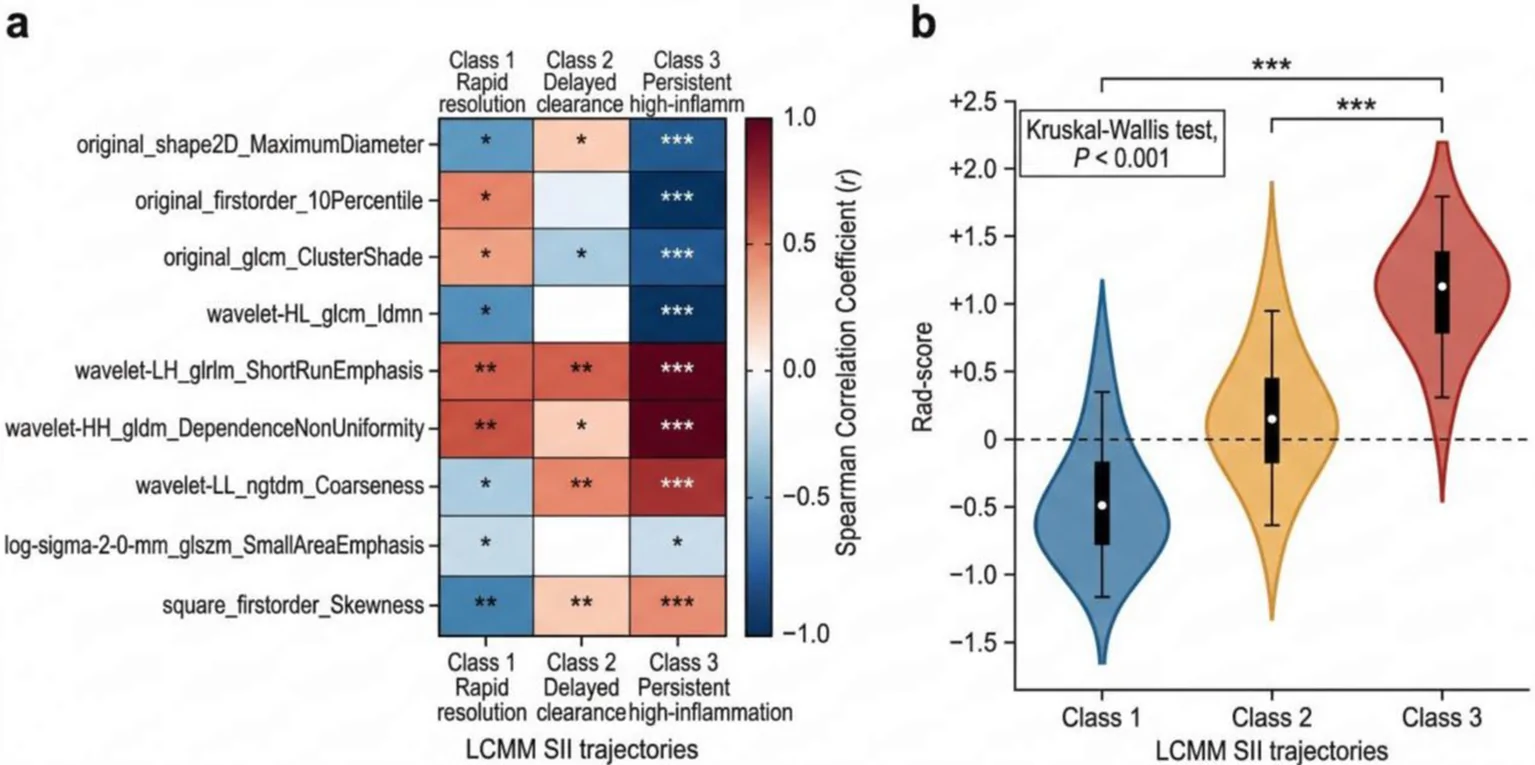
Cross-modal mapping between macroscopic upstream carotid radiomic architectures and microscopic downstream systemic immune trajectories. **(a)** A Spearman rank correlation matrix heatmap evaluating the association between the 9 core LASSO-derived radiomic features and the three LCMM longitudinal SII trajectories. The color gradient signifies the Spearman correlation coefficient (*r*), ranging from dark blue (strong negative correlation) to dark red (strong positive correlation). Statistical significance is denoted by asterisks (*p < 0.05, **p < 0.01, ***p < 0.001). Radiomic markers indicative of plaque vulnerability (e.g., highly fragmented textures like wavelet-LH_glrlm_ShortRunEmphasis) exhibited strong positive correlations specifically with the Class 3 hyper-inflammatory phenotype. **(b)** Violin plots illustrating the probability density distribution of the composite Carotid Ultrasound Rad-score, stratified by the three SII trajectory classes. Embedded internal boxplots display the median (white dot), interquartile range (thick vertical bar), and 1.5 × IQR (thin whiskers). Patients evolving into the “Persistent high-inflammation” trajectory (Class 3) harbored significantly higher baseline upstream Rad-scores compared to those in Class 1 and Class 2 (Kruskal-Wallis test, ***p < 0.001). LASSO, least absolute shrinkage and selection operator; SII, systemic immune-inflammation index; LCMM, latent class mixed model; iqr, interquartile range.

**Table 5:** Univariate and multivariate logistic regression analyses identifying independent predictors of 90-day unfavorable outcomes in the discovery cohort.

Variables	Unadjusted OR (95% CI)	P value	Adjusted OR (95% CI)	P value
Clinical and demographic factors				
Age (per 1-year increase)	1.05 (1.02–1.08)	0.003	1.03 (1.00–1.06)	0.042
Admission NIHSS score (per 1-point increase)	1.18 (1.12–1.25)	<0.001	1.14 (1.08–1.21)	<0.001
Diabetes mellitus (Yes vs. No)	1.85 (1.15–2.98)	0.012	1.42 (0.85–2.40)	0.175
Onset-to-puncture time (per 1-h increase)	1.12 (1.04–1.21)	0.004	1.08 (0.98–1.18)	0.115
Radiomic and longitudinal immune markers				
Carotid ultrasound Rad-score (per 0.1-point increase)	1.68 (1.38–2.06)	<0.001	1.52 (1.21–1.92)	<0.001
SII longitudinal trajectory				
Class 1 (Rapid resolution)	1.00 (Reference)	—	1.00 (Reference)	—
Class 2 (Delayed clearance)	2.85 (1.58–5.15)	<0.001	2.15 (1.12–4.15)	0.021
Class 3 (Persistent high-inflammation)	7.45 (3.65–15.40)	<0.001	4.85 (2.20–10.85)	<0.001

Because the plaque-to-embolus model is most plausible for atherosclerotic stroke, we performed an etiology-stratified sensitivity analysis. Among the 129 patients with large-artery atherosclerosis (TOAST-LAA), the Rad-score was a stronger independent predictor of unfavourable outcome (aOR 1.61; 95% CI, 1.24–2.09; *p* < 0.001) and the full multimodal model discriminated marginally better than in the overall cohort (AUC 0.93; 95% CI, 0.87–0.98). Among the 138 patients with cardioembolic stroke, the Rad-score remained significant but attenuated (aOR 1.38; 95% CI, 1.05–1.82; *p* = 0.021). The persistence of a weaker radiomic signal in cardioembolic stroke suggests that the ultrasound texture features index the patient’s systemic atherosclerotic and vascular-risk burden — a correlate of cerebrovascular reserve and reperfusion resilience — rather than functioning solely as a marker of the culprit embolus.

### 3.6 Development and validation of the multimodal machine learning model

To quantify the incremental predictive capability of the multimodal approach, four progressive models were developed: Model A (Clinical baseline), Model B (Clinical + Static baseline SII), Model C (Clinical + Ultrasound Rad-score), and Model D (Full Multimodal Model). Model D was engineered utilizing the Extreme Gradient Boosting (XGBoost) algorithm, integrating clinical variables, the Rad-score, and the categorical LCMM trajectory labels. Hyperparameter tuning (max_depth, learning_rate, n_estimators) was executed exclusively within the discovery cohort via grid search and 5-fold cross-validation (Supplementary Figure S2). Performance was subsequently evaluated in the strictly isolated internal validation cohort (*N* = 95). The optimal probability thresholds were derived utilizing the Youden Index solely from the discovery cohort and applied directly to the validation cohort to prevent data leakage. ROC curve analysis (Figures 6a,b) demonstrated that Model A achieved an AUC of 0.74 (95% CI, 0.63–0.84). The addition of the static baseline SII (Model B) yielded an AUC of 0.79 (95% CI, 0.69–0.88). The integration of radiomics (Model C) resulted in an AUC of 0.83 (95% CI, 0.74–0.91). Ultimately, the Full Multimodal XGBoost Model (Model D) achieved the highest discriminative performance, with an AUC of 0.92 (95% CI, 0.86–0.97) (Table 6). The non-parametric DeLong test confirmed that the AUC improvement of Model D over Model A was statistically significant (*p* < 0.001), whereas the difference between Model B and Model A was not (*p* = 0.125). Model D exhibited an Accuracy of 86.3%, Sensitivity of 88.0%, and Specificity of 84.4%. The calibration curve confirmed high concordance between the predicted probabilities and actual observed frequencies (Figure 6c).

**Figure 6:**
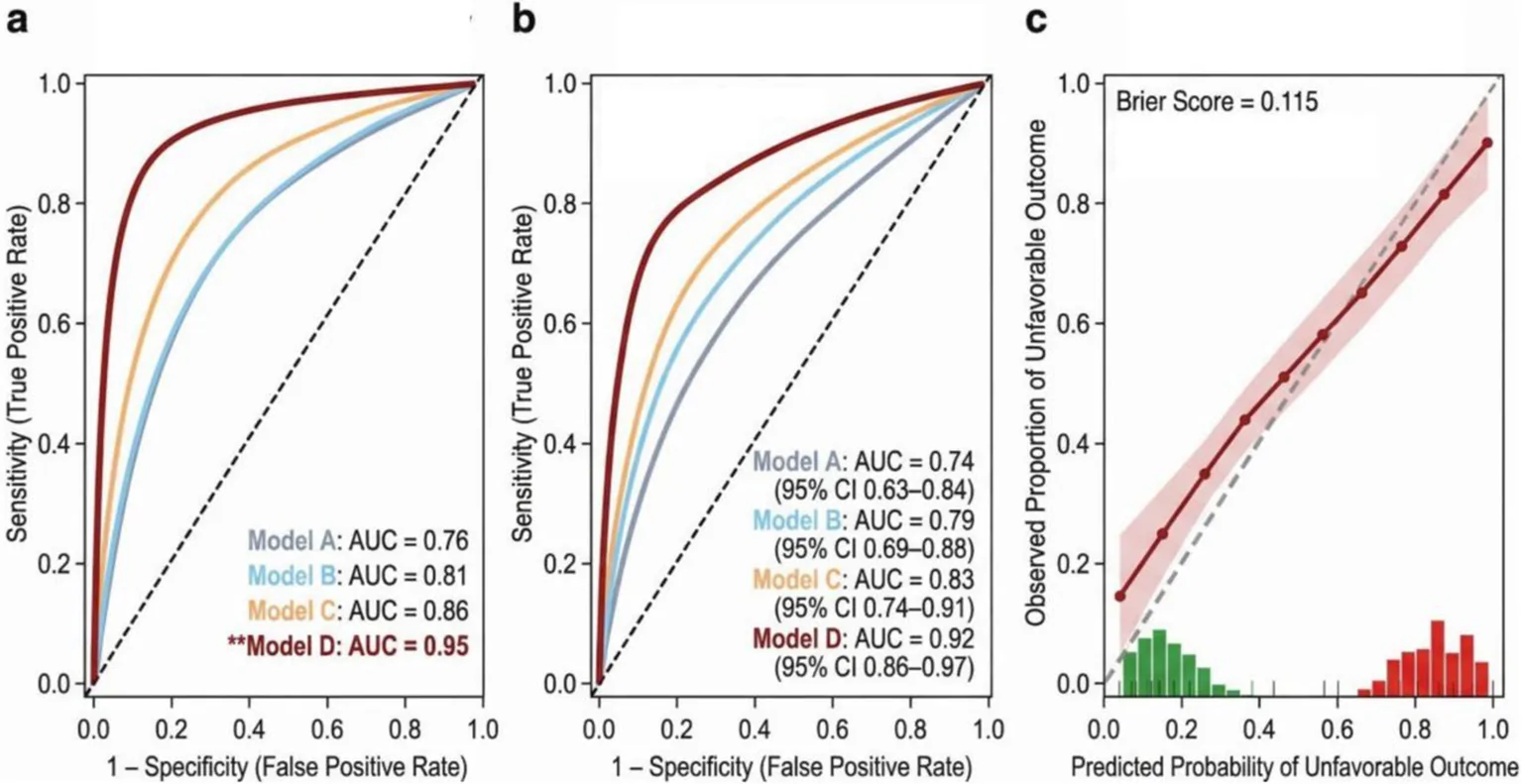
Independent validation and comparative performance evaluation of the multimodal machine learning models. **(a)** Receiver Operating Characteristic (ROC) curves demonstrating the discriminative performance of four progressive predictive models within the discovery cohort (*n* = 221): Standard clinical baseline (Model A), clinical + static SII (Model B), clinical + Rad-score (Model C), and the full multimodal Extreme Gradient Boosting (XGBoost) model integrating clinical, radiomic, and longitudinal LCMM data (Model D). The area under the ROC curve (AUC) is displayed for each configuration. **(b)** ROC curves evaluating identical models in the independent internal validation cohort (*n* = 95). The multimodal XGBoost model (Model D, crimson line) maintained a superior AUC of 0.92 (95% CI, 0.86–0.97). The superiority of Model D over the standard clinical Model A was statistically significant (DeLong test, *p* < 0.001). **(c)** Calibration curve assessing goodness-of-fit for the optimal Model D in the validation cohort. The *x*-axis represents predicted probability, and the *y*-axis represents observed proportion. The solid crimson line (with 95% CI shading) aligns closely with the ideal 45-degree reference line, supported by a low Brier score (0.115). The bottom histogram illustrates the density distribution of predicted probabilities. ROC, receiver operating characteristic; AUC, area under the curve; CI, confidence interval; XGBoost, extreme gradient boosting.

**Table 6:** Comprehensive performance evaluation of the four progressive machine learning models in the independent validation cohort.

Predictive models	AUC (95% CI)	*P* value*	Accuracy (%)	Sensitivity (%)	Specificity (%)	PPV (%)	NPV (%)
Model A: Clinical baseline alone	0.74 (0.63–0.84)	Reference	68.4	72	64.4	69.2	67.4
Model B: Clinical + Static baseline SII	0.79 (0.69–0.88)	0.125	73.7	76	71.1	74.5	72.7
Model C: Clinical + Ultrasound Rad-score	0.83 (0.74–0.91)	0.038	77.9	80	75.6	78.4	77.3
Model D: Full Multimodal XGBoost Model	0.92 (0.86–0.97)	<0.001	86.3	88	84.4	86.3	86.4

*P* values were calculated using the non-parametric DeLong test comparing the AUC of each model with that of Model A, the clinical baseline reference model.

Given the ratio of candidate predictors to events, several stability analyses were undertaken and the model is presented as exploratory. Bootstrap feature-selection stability (1,000 resamples) yielded a median selection frequency of 82.7% for the nine Rad-score features, with all wavelet-derived textural features selected in more than 80% of resamples (Supplementary Table S4). The LCMM classification was robust: the average maximum posterior probability was 0.89, the average posterior probability of assignment exceeded 0.85 for every class, and the odds of correct classification exceeded 7 for all classes. On repeated 10 × 10 nested cross-validation the full multimodal model achieved a mean AUC of 0.89 (SD 0.04), and bootstrap optimism correction gave a corrected AUC of 0.90 (apparent AUC 0.92; estimated optimism 0.02), indicating modest overfitting that reinforces the need for external validation.

To disentangle the contributions of data modality and learning algorithm, we performed a factorial comparison (Supplementary Table S5). Holding the algorithm fixed (XGBoost), discrimination rose from 0.76 (clinical only) to 0.86 (clinical + Rad-score) and 0.85 (clinical + trajectory), reaching 0.92 with all modalities combined, confirming that the added modalities — not the feature representation alone — drive the improvement. Holding the full feature set fixed, a penalised elastic-net logistic-regression benchmark achieved an AUC of 0.88 (95% CI, 0.81–0.94) and random forest 0.90 (95% CI, 0.84–0.96), neither differing significantly from XGBoost (DeLong *p* = 0.146 and *p* = 0.318, respectively). Thus, the gain attributable to gradient boosting over a parsimonious penalised regression using identical predictors was modest and non-significant; the substantive advantage is conferred by the multimodal integration itself.

To provide a stringent clinical benchmark, we constructed a comprehensive clinical/procedural model (Model A+) incorporating age, admission NIHSS, onset-to-puncture time, baseline ASPECTS, occlusion site, intravenous thrombolysis, puncture-to-recanalisation time, vascular risk factors, ASITN/SIR collateral grade, number of passes, first-pass effect, rescue therapy, and symptomatic intracranial haemorrhage. Model A + achieved an AUC of 0.81 (95% CI, 0.72–0.89), superior to the minimal clinical model (Model A, AUC 0.74). The full multimodal model nonetheless discriminated significantly better than this enhanced baseline (AUC 0.92 vs. 0.81; DeLong *p* = 0.011), with a continuous NRI of 0.612 (95% CI, 0.331–0.893; *p* < 0.001) and an IDI of 0.121 (95% CI, 0.074–0.168; *p* < 0.001). Model A + is therefore adopted as the primary clinical comparator throughout.

### 3.7 Incremental predictive value and clinical decision support

To precisely quantify the proportion of patients correctly reclassified by the multimodal paradigm, the Continuous Net Reclassification Improvement (NRI) and Integrated Discrimination Improvement (IDI) metrics were calculated (Table 7). When compared to the standard clinical baseline (Model A), the full multimodal approach (Model D) generated a continuous NRI of 0.985 (95% CI, 0.684–1.286; *p* < 0.001) and an IDI of 0.194 (95% CI, 0.128–0.260; *p* < 0.001). Furthermore, Model D demonstrated significant reclassification superiority even when compared directly against the radiomics-enhanced Model C (continuous NRI 0.520; 95% CI, 0.245–0.795; *p* < 0.001), validating the essential additive value of the spatiotemporal integration. Model interpretability was achieved via SHapley Additive exPlanations (SHAP) algorithms. The SHAP global summary plot (Figure 7a) ranked the feature importances driving the XGBoost predictions, verifying that the Class 3 SII longitudinal trajectory, the baseline NIHSS score, and specific wavelet-transformed carotid texture matrices possessed the highest predictive weights. Decision Curve Analysis (DCA) demonstrated that Model D provided a superior net clinical benefit across a threshold probability range of 10 to 85% compared to the reference models and the default “treat-all” or “treat-none” strategies (Figure 7b). To facilitate clinical translation, the parameters of the finalized multimodal model were utilized to construct an interactive, dynamic nomogram, allowing for individualized calculation of the absolute probability of an unfavorable 90-day functional outcome (Figure 7c).

**Table 7:** Incremental predictive value of the multimodal machine learning model assessed by continuous NRI and IDI in the validation cohort.

Reference model	Updated model	Continuous NRI (95% CI)	*P* value	IDI (95% CI)	*P* value
Model A	Model B	0.224 (−0.031–0.479)	0.085	0.038 (−0.002–0.078)	0.062
Model A	Model C	0.465 (0.198–0.732)	0.001	0.082 (0.035–0.129)	<0.001
Model A	Model D	0.985 (0.684–1.286)	<0.001	0.194 (0.128–0.260)	<0.001
Model B	Model D	0.761 (0.472–1.050)	<0.001	0.156 (0.102–0.210)	<0.001
Model C	Model D	0.520 (0.245–0.795)	<0.001	0.112 (0.068–0.156)	<0.001

Metric (Model D vs comparator)	vs Model A	vs Model A+	vs Model C
Continuous NRI (95% CI)	0.985 (0.684–1.286)	0.612 (0.331–0.893)	0.520 (0.245–0.795)
NRI events	0.542	0.348	0.291
NRI non-events	0.443	0.264	0.229
Categorical NRI (<30/30–70/>70%)	0.361 (0.198–0.524)	0.238 (0.101–0.375)	0.194 (0.062–0.326)
IDI (95% CI)	0.194 (0.128–0.260)	0.121 (0.074–0.168)	0.112 (0.068–0.156)
Calibration slope (95% CI)	0.94 (0.71–1.17)	—	—
Calibration intercept (95% CI)	−0.06 (−0.34 to 0.22)	—	—
Brier score (95% CI)	0.115 (0.083–0.147)	—	—

Reclassification, discrimination-improvement, and calibration metrics for the full multimodal model versus clinical baselines (validation cohort, *N* = 95).

Model A+, comprehensive clinical/procedural baseline. Reported as supportive exploratory evidence.

**Figure 7:**
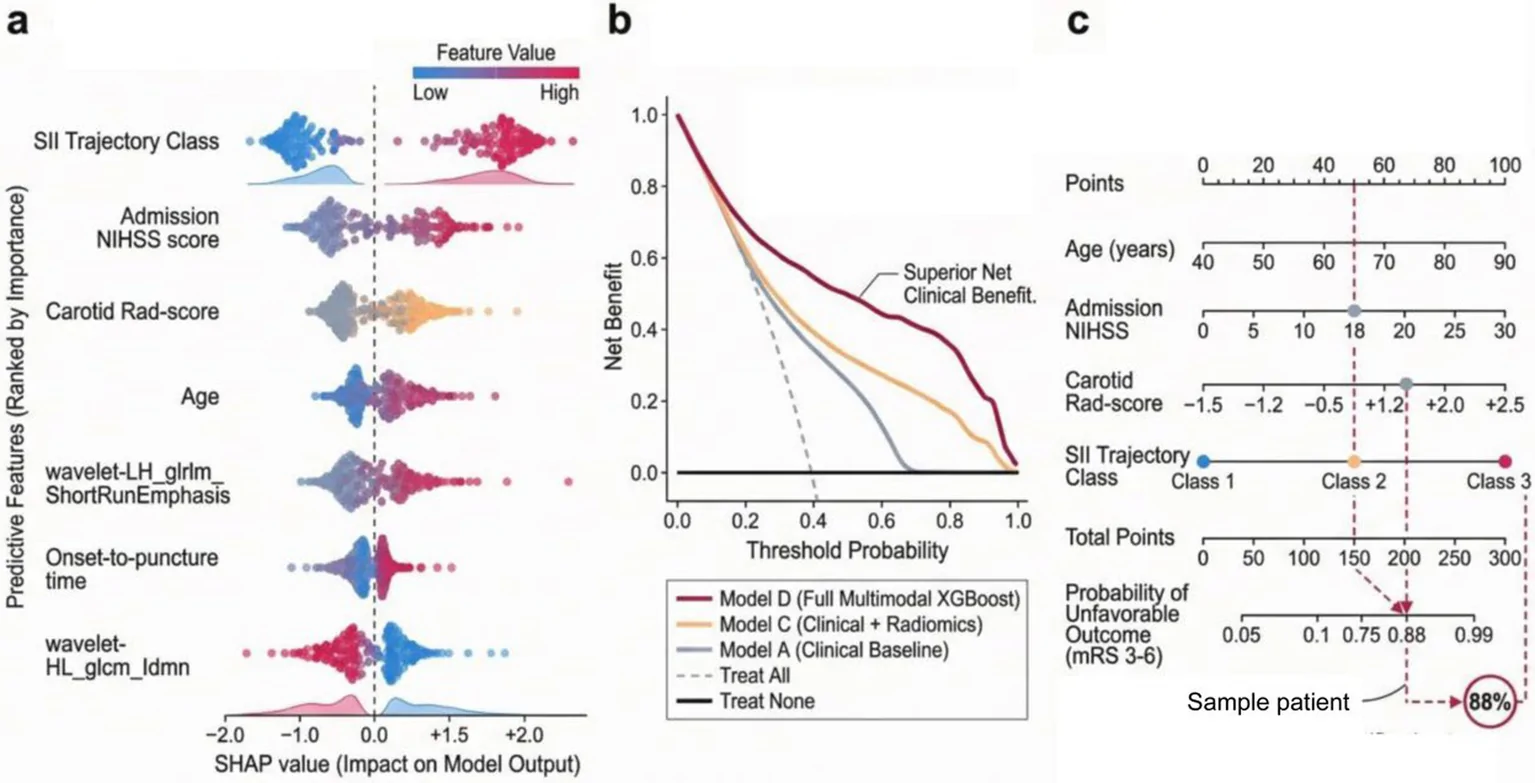
Explainable artificial intelligence (XAI) mapping and translation into an individualized clinical decision support system. **(a)** SHapley Additive Explanations (SHAP) global summary plot visualizing the inner decision-making logic of the optimal multimodal XGBoost model. The y-axis ranks predictive features in descending order of their mean absolute SHA*p* values. Each dot represents a single patient, positioned along the x-axis to denote the SHA*p* value (impact on model output), where values >0 indicate a driving force toward an unfavorable 90-day outcome. Color reflects the original feature value (Red = High, Blue = Low). Assignment to SII Trajectory Class 3, elevated admission NIHSS scores, and high Rad-scores were the most potent synergistic drivers of poor recovery. **(b)** Decision Curve Analysis (DCA) evaluating the clinical utility of the predictive models. The x-axis represents the threshold probability, and the y-axis represents net benefit. The full multimodal XGBoost model (Model D, crimson line) demonstrates superior net clinical benefit across a wide range of threshold probabilities (0.05 to 0.90) compared to alternative models and baseline assumptions (“Treat all” / “Treat none”). **(c)** A proof-of-concept interactive risk-display interface derived from the finalised exploratory model, provided to demonstrate feasibility only; it is not a validated bedside tool and requires prospective external validation, calibration monitoring, and usability testing before any clinical use. By mapping a specific patient’s values to the ‘Points’ axis and summing them to calculate ‘Total Points’, clinicians can determine the exact patient-specific probability of experiencing an unfavorable 90-day functional outcome (mRS 3–6). SHAP, shapley additive explanations; XGBoost, extreme gradient boosting; DCA, decision curve analysis; SII, systemic immune-inflammation index; NIHSS, National Institutes of Health Stroke Scale; mRS, modified Rankin Scale.

Reclassification metrics are reported as supportive, exploratory evidence rather than as proof of clinical utility. Relative to the comprehensive clinical baseline (Model A+), the full multimodal model yielded a continuous NRI of 0.612 (95% CI, 0.331–0.893; *p* < 0.001), decomposed into an event component of 0.348 and a non-event component of 0.264, and a categorical NRI across the pre-specified clinical strata (< 30% / 30–70% / > 70%) of 0.238 (95% CI, 0.101–0.375; *p* = 0.001); the IDI was 0.121 (95% CI, 0.074–0.168; *p* < 0.001). Calibration in the validation cohort was acceptable, with a slope of 0.94 (95% CI, 0.71–1.17), an intercept of −0.06 (95% CI, −0.34 to 0.22), and a Brier score of 0.115 (95% CI, 0.083–0.147). The wide confidence interval on the continuous NRI versus the minimal Model A (upper bound exceeding 1.0) reflects the known instability of this statistic in moderately sized samples and should be interpreted with caution.

## 4 Discussion

In this retrospective cohort study comprising 316 patients with AIS secondary to LVO treated with EVT, we developed and internally validated an exploratory multimodal machine-learning prognostic framework. The principal finding of this study is that the integration of macroscopic carotid ultrasound radiomics with the downstream micro-systemic longitudinal trajectories of the SII yields a highly accurate prediction of 90-day functional outcomes. The fully integrated XGBoost model (Model D) achieved an AUC of 0.92 (95% CI, 0.86–0.97) in the strictly isolated internal validation cohort, significantly outperforming the standard clinical baseline model (Model A, AUC 0.74; DeLong test *p* < 0.001) (Table 6). Furthermore, this multimodal paradigm provided reclassification utility, demonstrating a continuous NRI of 0.985 (95% CI, 0.684–1.286) and an IDI of 0.194 (95% CI, 0.128–0.260) (Table 7). These quantitative metrics substantiate the hypothesis that the spatiotemporal coupling of the upstream embolic source architecture and the temporal heterogeneity of the post-reperfusion thrombo-inflammatory storm is associated with neurological recovery.

The prognostic utility of the carotid ultrasound radiomic signature (Rad-score) reflects the histological complexity of the upstream atherosclerotic plaque. Out of 1,100 extracted features, the LASSO regression retained 9 core features, which remained an independent predictor of unfavorable outcomes (aOR, 1.52; 95% CI, 1.21–1.92 per 0.1-point increase) in the multivariate logistic regression (Table 5). Biologically, features exhibiting negative coefficients, such as original_firstorder_10Percentile (*β* = −0.315), directly quantify regions of hypoechogenicity. Prior ex vivo validation studies have established that such low-echogenicity zones strictly correspond to extensive lipid-rich necrotic cores and areas of intraplaque hemorrhage (21, 22). Conversely, positive coefficients for higher-order textural features like wavelet-LH_glrlm_ShortRunEmphasis (*β* = 0.287) denote high micro-textural fragmentation and spatial heterogeneity. Such acoustic heterogeneity is a well-documented surrogate for fibrous cap rupture and surface ulcerations (23). Our findings indicate that emboli originating from such structurally vulnerable, highly inflammatory plaques inherently possess greater histopathological rigidity and a more concentrated pro-inflammatory biochemical payload. When dislodged during EVT or spontaneous rupture, these specific embolic compositions exacerbate distal microvascular occlusion, increase the resistance to mechanical retrieval, and induce severe localized endothelial toxicity, leading to the “no-reflow” phenomenon and subsequent functional deterioration (24).

A central methodological feature of this study was the transition from static biomarker assessment to dynamic longitudinal profiling using LCMM. The post-EVT systemic immune response is not a monolithic event but a biphasic temporal cascade. The SII integrates the three principal cellular mediators of this cascade: neutrophils (driving acute blood–brain barrier disruption and oxidative stress via matrix metalloproteinases and neutrophil extracellular traps), platelets (mediating microvascular thrombosis and platelet–leukocyte aggregate formation), and lymphocytes (reflecting stroke-induced immunodepression and loss of neuroprotective regulatory T cells). By analysing the temporal variation of these components across Day 0, Day 1, Day 3, and Day 7, the LCMM identified three distinct trajectories (Figure 2b). The “persistent high-inflammation” trajectory (Class 3, 17.0% of the cohort) was independently associated with a near five-fold increase in the odds of 90-day unfavourable outcome (aOR 4.85; 95% CI, 2.20–10.85) relative to the “rapid resolution” trajectory (Class 1). Our cross-modal analysis revealed a moderate correlation between an elevated upstream Rad-score and the downstream Class 3 trajectory (Spearman *r* = 0.48; *p* < 0.001). We interpret this as a hypothesis-generating association rather than evidence of a causal “plaque-to-system” pathway: patients with texturally vulnerable plaques tend to carry a heavier systemic atherosclerotic and vascular-risk burden, greater baseline stroke severity, and a higher frequency of post-EVT complications, any of which could independently drive both the radiomic phenotype and the sustained inflammatory response. Consistent with this, the radiomic signal was stronger in large-artery atherosclerosis than in cardioembolic stroke. Because plaque histology, retrieved-thrombus histology, and formal mediation analysis were unavailable, the direction and mechanism of this association remain undetermined.

The imperative for precise prognostication following EVT has driven the development of numerous predictive models; however, the present study demonstrates substantial quantitative superiority over existing paradigms across multiple methodological dimensions. Initially, in the context of purely clinical predictive scores, the widely utilized Houston Intra-Arterial Recanalization Therapy (HIAR) score and the MR CLEAN registry nomogram have reported validation AUCs ranging between 0.72 and 0.76 (25, 26). Similarly, in our independent validation cohort, the standard clinical baseline model (Model A), which incorporated age, admission NIHSS, and onset-to-puncture time, achieved an AUC of 0.74 (95% CI, 0.63–0.84). The inherent limitation of these clinical models is their reliance on macroscopic, pre-intervention parameters, entirely neglecting the inter-individual heterogeneity in the biological response to the reperfusion injury itself. To address biological heterogeneity, recent literature has increasingly focused on peripheral inflammatory biomarkers. A large multicenter retrospective analysis by Zhang et al. (*N* = 543) demonstrated that a static, admission baseline SII ≥ 850 × 10^9/L predicted poor 3-month functional outcomes with an AUC of 0.69 (27). Consistent with their findings, our static baseline SII evaluation yielded an AUC of 0.71 (Table 2), and the integration of static SII into the clinical model (Model B) achieved an AUC of 0.79. However, static admission assessments suffer from a severe “ceiling effect.” The peripheral immune status at admission merely represents the baseline physiological state prior to the definitive immunological trigger—the endovascular reperfusion (28). Several recent investigations have attempted dynamic profiling using repeated measures of CRP or NLR. For instance, an analysis of the THRACE trial data indicated that a 48-h peak in NLR was associated with symptomatic intracranial hemorrhage, improving the predictive AUC from 0.73 to 0.78 (29). Our study fundamentally advances this concept by utilizing LCMM to cluster longitudinal SII data over a full 7-day post-operative window. By transforming continuous, chaotic longitudinal data into robust categorical phenotypic labels (Class 1, 2, and 3), the machine learning algorithm can effectively interpret the specific temporal “shape” of the inflammatory response. This methodological shift from static quantification to dynamic trajectory clustering is a primary driver of the performance leap observed in our Full Multimodal Model (Model D, AUC 0.92).

Notably, a penalised logistic-regression model using the same predictors performed comparably to XGBoost (AUC 0.88 vs. 0.92; *p* = 0.146), indicating that the incremental value reported here reflects the biological information contained in the combined radiomic and immune-trajectory features rather than the complexity of the machine-learning algorithm; a transparent penalised regression may therefore be preferable for future external validation.

Regarding radiomic analysis, the evaluation of carotid plaque vulnerability has predominantly been the domain of high-resolution magnetic resonance vessel wall imaging (HR-MR VWI) and CTA. A recent investigation utilizing HR-MR VWI radiomics in 182 AIS patients reported an excellent AUC of 0.85 for identifying symptomatic plaques (30). Similarly, CTA-based radiomics models have achieved AUCs of approximately 0.81 for predicting post-EVT hemorrhagic transformation (31). While highly accurate, MRI and advanced CTA are restricted by high costs, ionizing radiation (in CT), long acquisition times, and limited bedside availability in acute stroke settings. By leveraging standard two-dimensional B-mode ultrasound—a ubiquitous, non-ionizing, cost-effective, and point-of-care modality—our study to high-dimensional plaque phenotyping. The ultrasound-derived Rad-score, when integrated with clinical data (Model C), achieved an AUC of 0.83, demonstrating that filtered ultrasound textures (particularly via wavelet transformations) offer predictive capabilities highly comparable to those of advanced cross-sectional imaging modalities (32). Ultimately, the integration of these distinct data modalities via advanced machine learning differentiates this study from prior literature. Previous multimodal attempts in stroke neurology have primarily focused on combining clinical data with acute neuroimaging (e.g., CT perfusion parameters). A recent Random Forest model integrating clinical deficits with CT perfusion core-penumbra mismatch volumes reported an AUC of 0.86 (33). Another study utilizing an artificial neural network to combine clinical data with targeted plasma transcriptomics achieved an AUC of 0.89, albeit limited by a small sample size (*N* = 120) and the lack of external validation (34). Our XGBoost architecture (Model D) transcends these models by achieving an AUC of 0.92 and, more critically, an continuous NRI of 0.985 (*p* < 0.001) compared to the standard clinical model. In the domain of prognostic statistics, an NRI approaching 1.0 indicates a net reclassification benefit, although its wide confidence interval warrants cautious interpretation (35). This is consistent with the hypothesis that the combination of upstream macroscopic structural vulnerability (Radiomics) with downstream systemic micro-cellular evolution (LCMM SII Trajectories) creates a synergistic prognostic matrix that captures the full pathophysiological spectrum of post-EVT recovery far more comprehensively than any single-domain model (36).

The clinical implications of this multimodal framework are substantial. Currently, Phase III clinical trials evaluating post-EVT neuroprotective and immunomodulatory agents (e.g., natalizumab, fingolimod, and uric acid) have largely yielded neutral results (37, 38). A primary consensus for these failures is the lack of patient stratification; administering powerful immunomodulators to patients who naturally exhibit a “Rapid resolution” (Class 1) immune trajectory exposes them to severe infectious complications without providing therapeutic benefit (39). The LCMM trajectory phenotypes identified in our study provide an objective, data-driven criterion for clinical trial enrichment. Patients identified as possessing a high Rad-score and trending toward a Class 3 (“Persistent high-inflammation”) trajectory represent the precise target population that may benefit from aggressive, targeted anti-inflammatory interventions in the acute post-operative phase (40, 41). Furthermore, to bridge the gap between complex machine learning algorithms and routine clinical practice, we translated the parameters of the XGBoost model into an interactive dynamic nomogram (Figure 7c). This tool allows bedside clinicians to input standard clinical variables, the ultrasound Rad-score, and the patient’s early SII trajectory to rapidly compute the absolute probability of a 90-day unfavorable outcome, thereby facilitating data-driven decisions regarding intensive care unit triage, early rehabilitation intensity, and the necessity of prolonged neurological monitoring (42, 43).

## 5 Strengths and limitations

The primary strength of this study lies in its strict methodological adherence to rigorous validation standards. The use of a strictly isolated internal validation cohort, the prevention of data leakage through independent standardization, the requirement of high ICC values (>0.80) for radiomic feature retention, and the application of the DeLong test for AUC comparisons ensure the statistical robustness of the findings. Furthermore, the reliance on routinely acquired clinical data (ultrasound and complete blood counts) guarantees the high clinical feasibility and reproducibility of this model.

However, several limitations must be addressed. First, the retrospective, single-center design introduces inherent selection biases, despite the implementation of stratified random sampling. The predictive model must be subjected to external validation across multicenter cohorts with diverse ethnic and demographic backgrounds to definitively confirm its generalizability. Second, the segmentation of carotid ultrasound ROIs was performed manually. Although strict ICC thresholds were enforced to mitigate subjective variability, manual contouring is time-consuming and heavily operator-dependent. Future iterations of this model should integrate deep learning-based automated segmentation algorithms (e.g., U-Net architectures) to standardize the ROI extraction process and facilitate real-time, high-throughput analysis. Third, this study did not routinely incorporate advanced angiographic parameters such as the baseline collateral circulation status (e.g., American Society of Interventional and Therapeutic Neuroradiology/Society of Interventional Radiology [ASITN/SIR] grading) or the exact number of thrombectomy device passes, which are known to independently influence the final infarct volume and reperfusion injury severity. Finally, while the ultrasound radiomic features provide a highly accurate surrogate for plaque vulnerability, direct ex vivo histological validation of the plaques (via endarterectomy specimens) or the retrieved thrombi was not performed in this cohort, limiting our ability to definitively confirm the exact biochemical composition corresponding to the extracted wavelet matrices.

Additional limitations warrant emphasis. Because the SII trajectory is defined over a 7-day window, the “persistent high-inflammation” class is partly confounded by, and temporally entangled with, post-EVT complications such as pneumonia, symptomatic haemorrhage, and malignant oedema; although sensitivity analyses excluding or adjusting for these complications preserved a significant but attenuated association, the trajectory should not be interpreted as an outcome-independent immune biomarker, and reverse causation cannot be excluded in this observational design. Furthermore, the discovery cohort was below the calculated minimum development sample size; the model is therefore explicitly exploratory, and a penalised logistic-regression model using the same predictors performed comparably to gradient boosting, suggesting that a parsimonious, transparent model may be preferable for external validation.

## 6 Conclusion

In conclusion, we developed and internally validated an exploratory multimodal XGBoost model that integrates clinical variables, carotid plaque ultrasound radiomics, and LCMM-derived longitudinal trajectories of the Systemic Immune-Inflammation Index for subacute risk stratification of 90-day functional outcome in AIS-LVO patients after EVT. Because a key predictor matures only at Day 7, the framework is best positioned as a hypothesis-generating, post-procedural prognostic aid. External multicentre and prospective validation, together with prospective calibration monitoring, are required before the model can be considered for clinical use or for enrichment of interventional trials.

## Data Availability

The participant-level clinical and imaging data analyzed in this study are not publicly available because they contain potentially identifiable health information and are subject to institutional data-protection restrictions. De-identified data may be made available by the corresponding author upon reasonable request, subject to approval by the institutional ethics committee and applicable data-sharing requirements.
