# Supplementary material for: Multimodal integration of carotid plaque ultrasound radiomics and dynamic systemic immune-inflammation trajectories for subacute risk stratification after stroke thrombectomy: development and internal validation of an exploratory prognostic model
Source: Front Neurol. 2026 Oct 27;17:1896641. doi: 10.3389/fneur.2026.1896641 (PMC13747870; doi:10.3389/fneur.2026.1896641)
Supplement: Supplementary file 1 [file Supplementary_file_1.pdf]

Supplementary Figures

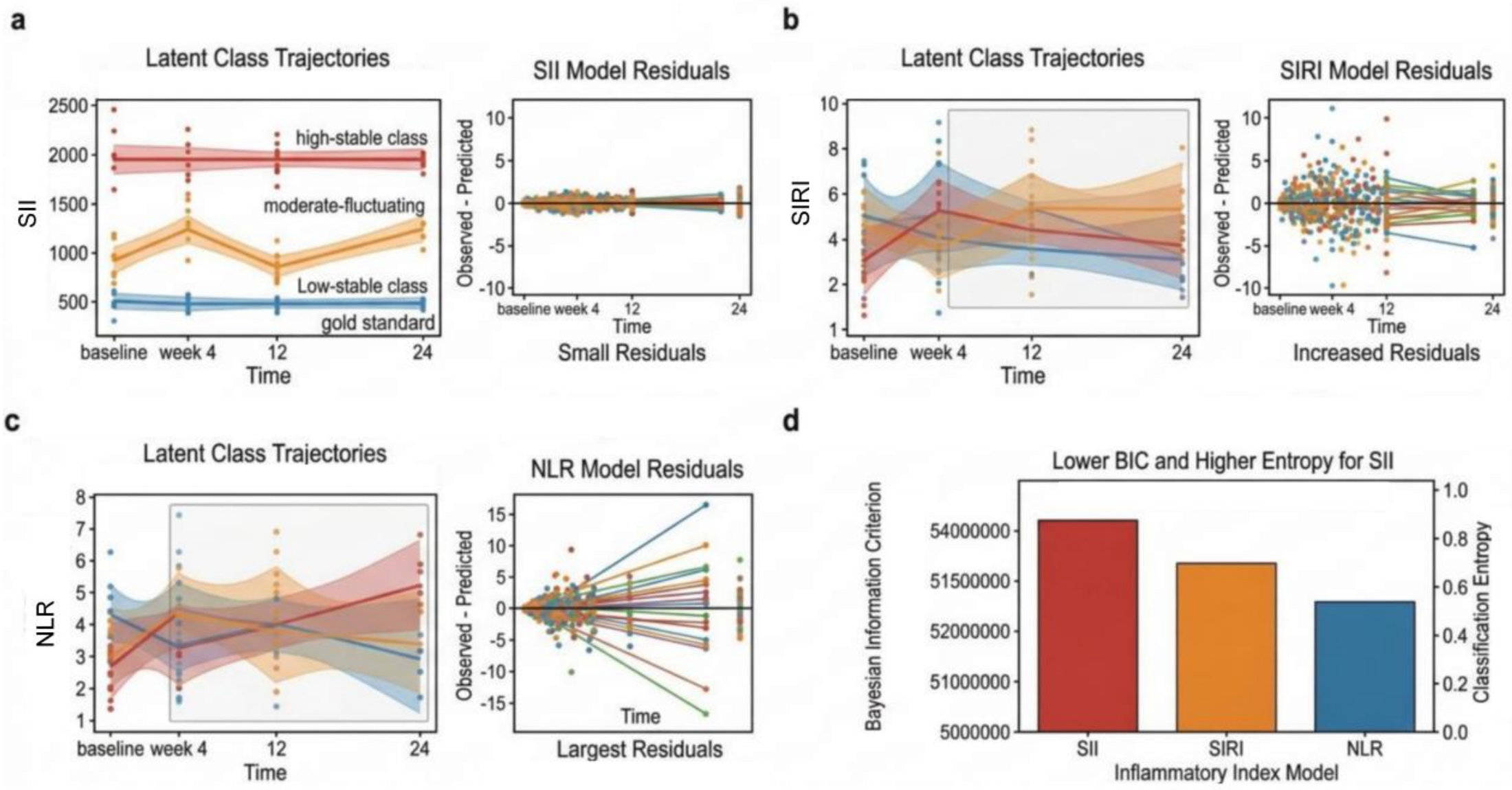

Supplementary Figure S1 | Sensitivity analysis of longitudinal trajectory modeling utilizing alternative systemic inflammatory indices. To justify the selection of the Systemic Immune-Inflammation Index (SII), parallel Latent Class Mixed Models (LCMM) specifying 3 latent classes were executed using the Systemic Inflammation Response Index (SIRI) and the Neutrophil-to-Lymphocyte Ratio (NLR). **a**, The reference LCMM trajectory model utilizing transformed SII data, demonstrating excellent trajectory divergence and tight 95% Confidence Intervals (CI). The inset confirms tightly distributed, homoscedastic subject-specific residuals. **b**, LCMM modeling utilizing transformed SIRI data. Severe trajectory overlap and excessively wide CIs indicate classification uncertainty. The inset residual plot reveals wide dispersion. **c**, LCMM modeling utilizing transformed NLR data. Trajectories fail to adequately isolate the extreme hyper-inflammatory phenotype observed in the SII model. **d**, Quantitative goodness-of-fit comparison across the three biomarker models. The SII model achieved a classification Entropy of 0.88 (exceeding the 0.80 optimality threshold), whereas the SIRI (0.65) and NLR (0.61) models exhibited poor class separation. Furthermore, the SIRI and NLR models yielded substantially inferior Bayesian Information Criterion (BIC) values (ΔBIC = +145.2 and +210.8, respectively) compared to the optimal SII model. Abbreviations: LCMM, Latent Class Mixed Model; SII, Systemic Immune-Inflammation Index; SIRI, Systemic Inflammation Response Index; NLR, Neutrophil-to-Lymphocyte Ratio; BIC, Bayesian Information Criterion; CI, Confidence Interval.

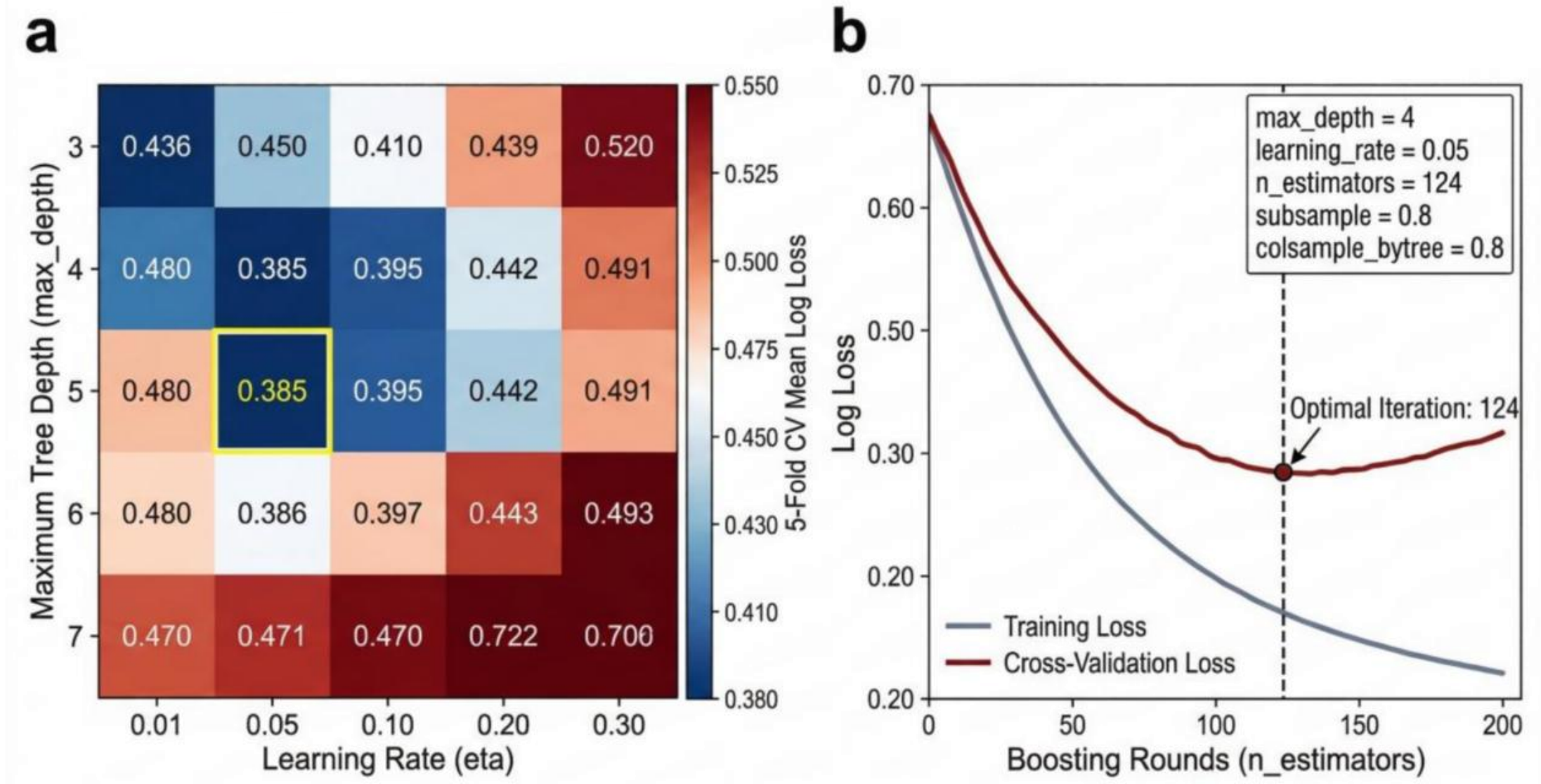

Supplementary Figure S2 | Hyperparameter tuning and convergence optimization of the multimodal Extreme Gradient Boosting (XGBoost) model. **a**, A grid search heatmap visualizing the 5-fold cross-validation logarithmic loss (Log Loss) landscape across combinations of two critical hyperparameters: learning_rate and max_depth (tree depth). The color gradient from deep blue (optimal, low error) to dark red (suboptimal, high error) maps the error distribution. The optimal spatial configuration, yielding the global minimum cross-validation Log Loss (0.385), was locked at a max_depth of 4 and a learning_rate of 0.05, indicated by the yellow-bordered cell. **b**, Optimization convergence and early stopping learning curves for the locked model configuration. The x-axis denotes the number of sequential boosting rounds (n_estimators), while the y-axis represents the corresponding Log Loss. While the training loss (slate gray line) continuously reduces, the cross-validation loss (crimson line) reaches its global minimum at exactly 124 boosting rounds. The implementation of an early stopping callback terminated the algorithm at this inflection point, locking the final configuration to ensure maximal generalization performance and actively prevent overfitting in the validation cohort. Abbreviations: XGBoost, Extreme Gradient Boosting; CV, Cross-Validation.

Supplementary Tables

Supplementary Table S1. IBSI-compliant radiomics image processing and feature extraction parameter checklist.

Parameter Category	Parameter Details	Specific Values / Settings applied in this study
1. Image Data & Software	Modality	2D Carotid Ultrasound (B-mode)
	Image format	DICOM converted to NIfTI (.nii.gz)
	Extraction software & Version	PyRadiomics (version 3.0.1) in Python 3.9.7
	IBSI compliance	Yes (PyRadiomics is an IBSI-compliant software)
2. ROI Segmentation	Segmentation method	Manual delineation, blinded to clinical outcomes
	Software used for ROI	ITK-SNAP (version 3.8.0)
	ROI dimensionality	2D (Carotid plaque or thickened intima-media)
3. Image Interpolation	Resampling method	2D spatial resampling applied to all images
	Resampled pixel spacing	1.0 mm × 1.0 mm
	Interpolator (Image)	sitkBSpline (B-spline interpolation)
	Interpolator (ROI Mask)	sitkNearestNeighbor (Nearest Neighbor interpolation)
	Pad distance	5 pixels
4. Image Discretization	Discretization method	Fixed Bin Width (FBW)
	Bin width size	25
	Lowest intensity bound	None (Determined by image minimum)
5. Feature Extraction	Dimensions	force2D = True, force2Ddimension = 0
	Distance weighting	Chebyshev norm (Distance = 1 pixel)
	Symmetrical GLCM	TRUE
	Feature classes extracted	Shape2D, First-order, GLCM, GLRLM, GLSZM, GLDM, NGTDM
6. Image Filters	Original	Applied (No filter)
	Wavelet (2D)	Applied (Channels: LL, LH, HL, HH)
	Laplacian of Gaussian (LoG)	Applied (σ = 2.0, 3.0, 4.0 mm)
	Additional filters	Square, SquareRoot, Logarithm, Exponential

Supplementary Table S2. Top 30 radiomic features demonstrating high intra- and inter-observer reproducibility (ICC > 0.80).

Radiomic Feature (IBSI Nomenclature)	Intra-observer ICC (95% CI)	Inter-observer ICC (95% CI)
original_shape2D_MaximumDiameter	0.982 (0.971–0.989)	0.965 (0.945–0.978)
original_shape2D_MeshSurface	0.978 (0.965–0.987)	0.958 (0.932–0.973)
original_shape2D_Sphericity	0.956 (0.931–0.972)	0.941 (0.910–0.962)
original_firstorder_10Percentile	0.965 (0.942–0.978)	0.944 (0.912–0.965)
original_firstorder_Entropy	0.942 (0.911–0.963)	0.925 (0.885–0.952)
original_firstorder_Skewness	0.935 (0.898–0.958)	0.912 (0.870–0.942)
original_firstorder_Uniformity	0.951 (0.925–0.969)	0.933 (0.895–0.957)
original_glcm_ClusterShade	0.918 (0.875–0.946)	0.892 (0.840–0.928)
original_glcm_Idmn	0.905 (0.852–0.938)	0.885 (0.825–0.924)
original_glcm_Contrast	0.922 (0.881–0.950)	0.901 (0.845–0.936)
original_glrlm_ShortRunEmphasis	0.938 (0.905–0.960)	0.915 (0.872–0.944)
original_glszm_SmallAreaEmphasis	0.927 (0.886–0.953)	0.904 (0.850–0.938)
wavelet-LL_firstorder_Mean	0.958 (0.935–0.974)	0.942 (0.908–0.964)
wavelet-LL_glcm_Correlation	0.912 (0.865–0.942)	0.886 (0.828–0.925)
wavelet-HL_glcm_Idmn	0.895 (0.840–0.931)	0.872 (0.810–0.915)
wavelet-LH_glrlm_ShortRunEmphasis	0.908 (0.858–0.940)	0.884 (0.822–0.924)
wavelet-HH_gldm_DependenceNonUniformity	0.885 (0.825–0.924)	0.862 (0.795–0.908)
wavelet-LL_ngtdm_Coarseness	0.892 (0.835–0.929)	0.875 (0.812–0.916)
log-sigma-2-0-mm_glszm_SmallAreaEmphasis	0.915 (0.870–0.944)	0.890 (0.835–0.927)
square_firstorder_Skewness	0.928 (0.888–0.952)	0.905 (0.852–0.938)
wavelet-HL_glrlm_RunLengthNonUniformity	0.888 (0.830–0.926)	0.865 (0.800–0.910)
wavelet-LH_glcm_Autocorrelation	0.902 (0.850–0.935)	0.880 (0.818–0.921)
wavelet-HH_firstorder_Kurtosis	0.910 (0.862–0.941)	0.885 (0.825–0.925)
log-sigma-3-0-mm_glcm_Imc1	0.894 (0.838–0.930)	0.870 (0.805–0.914)
log-sigma-4-0-mm_glszm_ZoneEntropy	0.882 (0.818–0.922)	0.858 (0.785–0.905)
wavelet-LL_gldm_HighGrayLevelEmphasis	0.920 (0.878–0.948)	0.896 (0.842–0.932)
wavelet-HL_ngtdm_Contrast	0.875 (0.805–0.918)	0.852 (0.775–0.900)
square_glcm_JointEnergy	0.932 (0.895–0.956)	0.912 (0.865–0.942)
squareroot_firstorder_Median	0.955 (0.928–0.971)	0.938 (0.902–0.960)
logarithm_glrlm_LongRunEmphasis	0.906 (0.855–0.938)	0.882 (0.820–0.922)

Supplementary Table S3. Distribution of post-EVT complications and in-hospital treatment exposures across LCMM-derived SII trajectory classes.

Complication / exposure	Overall (N=316)	Class 1 (n=120)	Class 2 (n=142)	Class 3 (n=54)	P*
Stroke-associated pneumonia	68 (21.5%)	12 (10.0%)	31 (21.8%)	25 (46.3%)	<0.001
Symptomatic ICH (Heidelberg)	23 (7.3%)	4 (3.3%)	10 (7.0%)	9 (16.7%)	0.006
Any haemorrhagic transformation	104 (32.9%)	26 (21.7%)	48 (33.8%)	30 (55.6%)	<0.001
Malignant cerebral oedema	41 (13.0%)	7 (5.8%)	18 (12.7%)	16 (29.6%)	<0.001
Non-pulmonary systemic infection	47 (14.9%)	11 (9.2%)	21 (14.8%)	15 (27.8%)	0.004
ICU admission	98 (31.0%)	24 (20.0%)	46 (32.4%)	28 (51.9%)	<0.001
Systemic antibiotic exposure	89 (28.2%)	18 (15.0%)	40 (28.2%)	31 (57.4%)	<0.001
Corticosteroid exposure	34 (10.8%)	6 (5.0%)	15 (10.6%)	13 (24.1%)	0.001

**Chi-square or Fisher's exact test across the three classes. ICH, intracranial haemorrhage; ICU, intensive care unit.*

Supplementary Table S4. Bootstrap selection frequency of the nine Rad-score features across 1,000 resamples of the discovery cohort.

Radiomic feature	Bootstrap selection frequency (%)
wavelet-HL_glcm_Idmn	94.2
original_firstorder_10Percentile	91.8
wavelet-LH_glrlm_ShortRunEmphasis	88.5
log-sigma-2-0-mm_glszm_SmallAreaEmphasis	86.1
wavelet-HH_gldm_DependenceNonUniformity	82.7
wavelet-LL_ngtdm_Coarseness	80.3
original_glcm_ClusterShade	78.6
original_shape2D_MaximumDiameter	74.9
square_firstorder_Skewness	71.2

Supplementary Table S5. Discrimination of comparator models in the internal validation cohort (N = 95), separating the contribution of data modality from that of the learning algorithm.

**Model (feature set / algorithm)**	**AUC (95% CI)**	**vs XGBoost-full (DeLong P)**
**Same algorithm (XGBoost), varying features:**		
Clinical only	0.76 (0.66–0.85)	<0.001
Clinical + Rad-score	0.86 (0.79–0.93)	0.041
Clinical + SII trajectory	0.85 (0.77–0.92)	0.036
**Same features (full), varying algorithm:**		
Penalised (elastic-net) logistic regression	0.88 (0.81–0.94)	0.146
Random forest	0.90 (0.84–0.96)	0.318
XGBoost (Model D, reference)	0.92 (0.86–0.97)	—
